# Endocannabinoid system and phytocannabinoids in the main species of veterinary interest: a comparative review

**DOI:** 10.1007/s11259-024-10509-7

**Published:** 2024-08-20

**Authors:** Alessandra Di Salvo, Elisabetta Chiaradia, Monica Sforna, Giorgia della Rocca

**Affiliations:** 1 Department of Veterinary Medicine, University of Perugia, Perugia, Italy; 2 Research Center on Animal Pain (CeRiDA), University of Perugia, Perugia, Italy

**Keywords:** ECS, Phytocannabinoids, Dog, Cat, Horse

## Abstract

Since the discovery of the endocannabinoid system and due to the empirical evidence of the therapeutic effects on several illnesses both in humans and animals that follow the administration of exogenous cannabinoids (i.e., phytocannabinoids), numerous studies have been conducted. These investigations aimed to identify the expression and distribution of cannabinoid receptors in healthy and pathologic organs and tissues of different animal species and to define the interactions of phytocannabinoids with these receptors. In the last decade, pharmacokinetics, efficacy and tolerability of many Cannabis derivatives formulations, mainly containing cannabidiol, in the main species of veterinary interest, have been also investigated. This manuscript summarizes the findings reported by the scientific studies published so far on the molecular mode of action of the main phytocannabinoids, the localization of cannabinoid receptors in organs and tissues, as well as the pharmacokinetics, efficacy and tolerability of Cannabis derivatives in dogs, cats, horses and other species of veterinary interest. A deep knowledge of these issues is crucial for the use of phytocannabinoids for therapeutic purposes in animal species.

## Introduction

The endocannabinoid system (ECS) is a sophisticated and intricate signalling network involved in different biological processes, within both neural and non-neural tissues. It has been described as maintaining homeostatic equilibrium in response to environmental factors and metabolic stress conditions (Lutz 2020).

The ECS can be conceptually defined as a network comprising cannabinoid receptors such as cannabinoid receptor type 1 (CB1), cannabinoid receptor type 2 (CB2), G protein-coupled receptor 55 and 119 (GPR55, GPR119), and related receptors such as transient receptor potential vanilloid (TRPV) and peroxisome proliferator-activated receptor (PPAR). It also includes endocannabinoids (eCBs) such as anandamide (AEA) and 2*-*Arachidonoylglycerol (2-AG), endocannabinoid-like signaling molecules, proteins involved in their transport, enzymes responsible for endocannabinoid synthesis and catabolism, and the genes encoding these proteins (Kibret et al. 2023; Migliaro et al. 2023). Nevertheless, offering a detailed explanation of the ECS remains difficult due to the ongoing discovery and development of its potential components, receptors, and pathways.

Coined as the “endocannabinoidome” (Di Marzo and Wang 2015; Fraguas-Sánchez et al. 2018), this system plays a vital role in maintaining homeostatic functions, exhibiting antioxidant, hypotensive, immunosuppressive, anti-inflammatory, and pain-relieving effects. The distribution of cannabinoid receptors in the brain hints at physiological involvement in movement control, perception, sleep, appetite regulation, inhibition of learning and memory, emotional state regulation, and neuroprotection. The ECS affects vasomotor function, fertility, and even tumor cell proliferation (Fraguas‐Sánchez et al. 2018).

Knowledge of this intrinsic system prompted exploration into how active ingredients, particularly phytocannabinoids (pCBs) contained in *Cannabis sativa*, interact with it, leading to both therapeutic and psychotropic effects. In veterinary medicine, consideration of *Cannabis* derivatives for therapeutic purposes emerged a few years ago. Potential applications include pain management, neurological conditions, well-being, gastrointestinal health, dermatologic diseases, and oncology (Di Salvo et al. 2023). With growing awareness of the therapeutic potential of *Cannabis* derivatives in veterinary medicine and the legalization of cannabinoids in some states, veterinarians and pet owners are increasingly exploring cannabinoid products for their companion animals (Kogan et al. 2019a, b). Indeed, according to the analysis carried out by Zion Market Research, the CBD pet market was estimated to be about 257.6 Million US dollars in 2023, and it is expected to increase up to 2,967.40 Million US dollars at the end of 2032 (https://www.zionmarketresearch.com/report/cbd-pet-market; accessed 15 July 2024).

This paper aims to review the molecular mechanisms by which pCBs carry out their physiological and pharmacological actions and to summarize data published so far about the expression of the ECS and the pharmacokinetics, efficacy, and tolerability of *Cannabis* derivatives in the main species of veterinary interest.

## Mode of action of phytocannabinoids

Based on their sources, cannabinoids can be classified as eCBs that are specifically synthesised in animal cells, pCBs mainly found in cannabis plant, and synthetic cannabinoids (sCBs), designed to mimic the effects of natural cannabinoids. Endocannabinoids and pCBs have both hydrophobic proprieties but different chemical structures. The eCBs are endogenous lipid compounds composed of a long-chain polyunsaturated fatty acid tail and a polar head containing functional groups like amide, ester, ether, or hydroxy groups, while pCBs are terpenophenolic substances containing tricyclic, bicyclic, and monocyclic structures that can exists in different isomers (Maccarrone et al. 2023). Although eCBs and pCBs differ in their chemical structures, they exhibit overlapping effects, although with some variability, through target receptors, signaling pathways, and enzymes involved in their metabolism. Indeed, the ECS was originally identified by studying the mechanism of action of the psychotropic cannabis substance, Δ9 -tetrahydrocannabinol (THC) (Silver 2019) which is the major components of the *Cannabis* plant extract. Meanwhile, cannabidiol (CBD) represent the most abundant non-psychoactive compound of *Cannabis* plants. Further, other active compounds including cannabigerol (CBG), Cannabigerovarin (CBGV), cannabichromene (CBC), cannabielsoin (CBE), cannabicyclol (CBL), cannabinol (CBN), (CBC), cannabinodiol (CBND), cannabitriol (CBT), cannabidivarin (CBDV), tetrahydrocannabivarin (THCV), as well as their analogues (such as Δ8-THC and Δ10-THC, among others), their acid derivatives (THCA, CBDA, CBGA, CBGVA, CBCA, CBDVA, etc.) and other minor cannabinoids, terpenes, and flavonoids have been extracted from different *Cannabis* species (Maccarrone et al. 2023; Mechoulam 2023). This plethora of bioactive molecules makes it difficult to define the specific effects of *Cannabis* extracts but provides “lead” molecules for therapeutic and medical applications. Thus, the understanding of pharmacological properties and potential effects of pCBs is still superficial and often inferred from findings obtained in studies primarily focused on the pathways activated by the interaction between eCBs and CB receptors (Maccarrone et al. 2023; Mechoulam 2023). Nevertheless, it is accepted that pCBs act by binding specific receptors of eCBs in animal cells and by imitating their effects. Most of the findings reviewed in this section have been extrapolated from mouse and human studies.

**Fig. 1:**
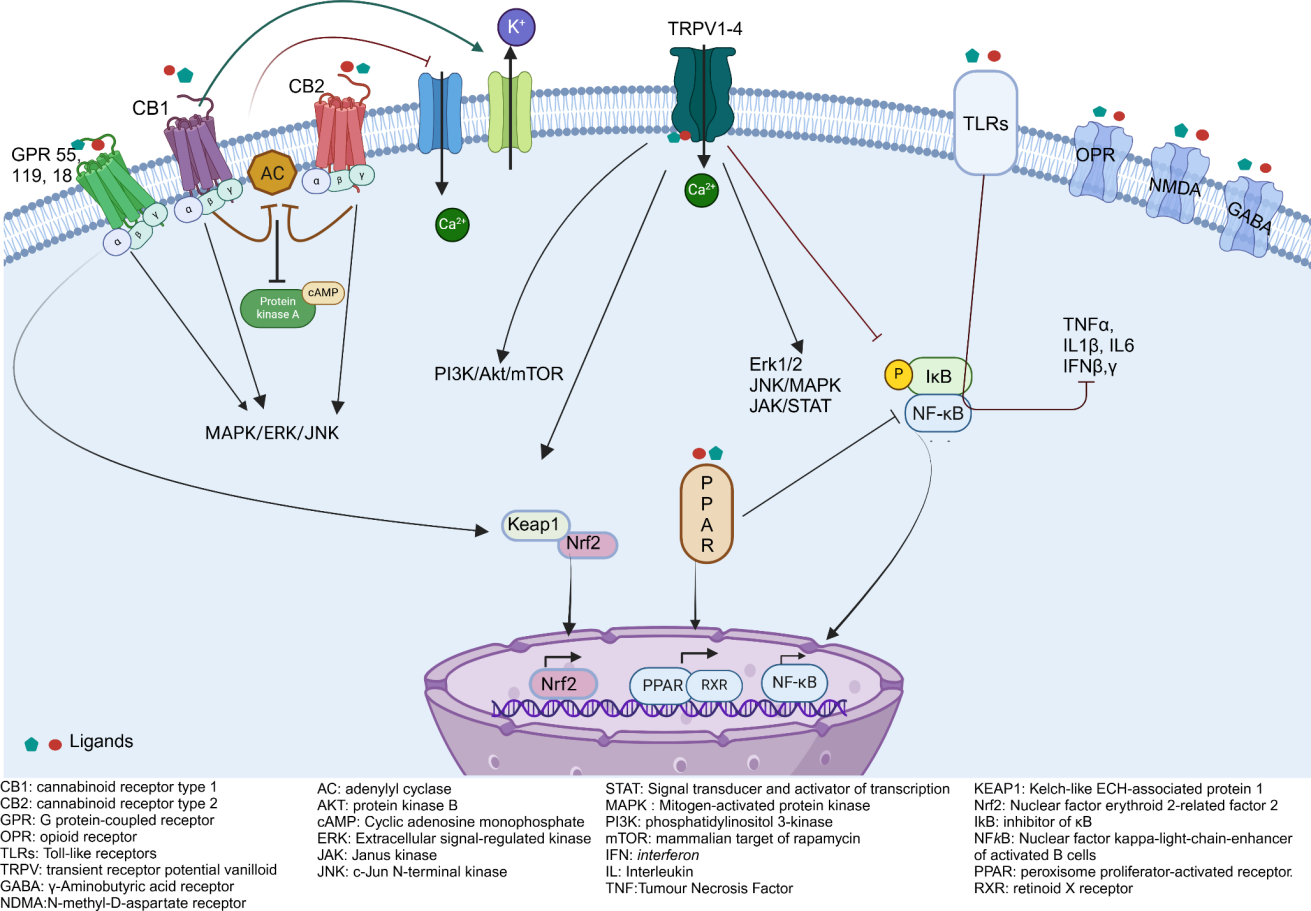
Schematic representation of the downstream pathways of cannabinoid receptors described in the text. (Created with BioRender.com)

Cannabinoids may engage with various receptors including: G protein-coupled receptors (GPCRs) such as CB1, CB2, GPR55, GPR119, and GPR18, that are transmembrane proteins with an extracellular binding; Transient Receptor Potential Vanilloid (TRPV) 1, 2, 3, 4 also located on the plasma membrane but having intracellular binding sites; nuclear PPARs receptors by which cannabinoids may regulate gene expression (Gomez-Canas et al. 2023) (Fig. 1). Further, CB1 has been described in the mitochondrial membrane. The ability to interact with various receptors with different downstream pathways is considered the basis for the different, sometimes opposite effects of various pCBs (Maccarrone et al. 2023).

### G protein-coupled receptors (GPCRs)

Cannabinoid receptor 1 (CB1) and cannabinoid receptor 2 (CB2) (numbered in the order of their discovery) represent the most extensively investigated cannabinoid receptors. CB1 and CB2 differ in tissue distribution, affinity and intrinsic activity to the various pCBs. In particular, THC has a high affinity for CB1 compared to CBD, which interacts mainly with CB2; CBD may also act as a negative allosteric modulator of CB1. CBG and CBC have a relatively low affinity for CB1 and CB2 receptors, while CBN has been considered a partial agonist at CB1 receptors. Surprisingly, even if THCV has a structure like THC, it everts a higher affinity for CB2 than CB1 (McPartland et al. 2015), The ability to recognize different receptors with different affinities results into different effects of pCBs, that also reflect the specific cellular and tissue localization of these receptors (Blebea et al. 2024). Overall, the CB1 receptor is widely expressed in the brain, primarily localized at the presynaptic axon terminals (both excitatory glutamatergic and inhibitory γ-amino-butyric acid (GABA)-ergic) of neurons, as well as in astrocytes and microglia; CB2 receptors are predominantly found in immune system cell components (as monocyte-derived cells and lymphocytes) and in peripheral tissues including endothelial cells, lungs, tonsils, enteric neurons, synovial membrane, and skin keratinocyte (Bie et al. 2018; Zou 2018). Moreover, CB2 receptors have been observed within the central nervous system (CNS) such as the amygdala, hippocampus, cerebellum, cerebral cortex, striatum, etc. Similarly, CB1 has been detected in peripheral tissues including the liver, pancreas, small intestine, and skeletal muscle (Lim et al. 2023; Miranda-Cortés et al. 2023). Species specific localization of receptors is described in the next paragraph. The activation of both CB1 and CB2, coupled with G proteins (mainly Gi/o-types), leads to inhibition of the adenylyl cyclase activity with reduction of cellular cyclic adenosine monophosphate (cAMP) levels and protein kinase A (PKA) activity (Ye et al. 2019; Maccarrone et al. 2023). Moreover, CB1 receptors are also coupled, via G proteins, to potassium channels, and their activation is followed by the hyperpolarization of postsynaptic neurons (Irving et al. 2017; Maccarrone et al. 2023). CB1 and CB2 stimulation can also lead to the activation of mitogen-activated protein kinase (MAPK) signaling pathways, extracellular kinase-1 and − 2 (ERK1/2), p38 and p42/p44 MAPKs, and c-Jun N-terminal kinase (JNK), involved in the regulation of cell proliferation and death.

The activation of MAP/ERK/JNK pathways by pCBs can also be mediated via GPR55, GPR119, and GPR18 (Irving et al. 2017). Even if GPR55 endogenous ligand is mainly the phospholipid lysophosphatidylinositol (LPI), CBD has been reported as its potent antagonist (Ryberg et al. 2007; Whyte et al. 2009). GPR55 has been evidenced in the brain, in the peripheral system and in various tissues co-localized with CB1 and CB2. GPR55 was found associated with different G-proteins (Gαq/11, Gα12, Gα13, or Gα12/13) in the stimulation of intracellular Ca^2+^ release through Rho signalling pathway (Whyte et al. 2009; Morales and Reggio 2017). However, the activation of GPR55 can have also effects on the antioxidant response mediated by nuclear factor erythroid 2-related factor 2 (Nrf2) and ERK pathways (Irving et al. 2017). Cannabidiol has also been proposed as an antagonist of GPR18, widespread in almost all tissues (Morales et al. 2020; Senn et al. 2020).). The activation of GPR18 by its endogenous ligand N-arachidonoyl glycine (NAGly) can induce intracellular Ca^2+^ mobilization (Console-Bram et al. 2014). Similar effects can be exerted by THC via GPR18, in a dose dependent manner (Senn et al. 2020). Otherwise, CBD can block/reduce the effects of both NAGly and THC on GPR18 (Irving et al. 2017). These receptors seem to be also involved in the effects of pCBs and metabolic pathways via insulin signalling and cAMP (Soga et al. 2005).

### Transient receptor potential vanilloid (TRPV)

The anti-hyperalgesic effects of CBD, and in some cases its anti-inflammatory properties, appear to be mediated by TRPV1 (Etemad et al. 2022). Phitocannabinoids such as CBD, THC and CBN, CBG, CBC and Δ9-THCV show different TRPV type 1–4 agonistic activities (Etemad et al. 2022). Overall, TRPVs are non-selective cation channels allowing calcium influx into various types of cells (Huang et al. 2024). TRPV1 may be activated by CBD and minor cannabinoids but not by Δ9-THC (Starkus et al. 2019), which activates TRPV2. Cannabidiol has high potential to bind TRPV1 and with slight differences TRPV2 as well as TRPV3, TRPV4, and transient receptor potential ankyrin subtype 1 (TRPA1) (Etemad et al. 2022). Working as a TRPV1 potent agonists, CBD induces a rapid desensitization of TRPV1 channels, leading to a decrease in calcium influx (Etemad et al. 2022). Indeed, CBD-induced anti-hyperalgesia was nullified by the TRPV1 antagonist capsazepine but not by CB1 or CB2 antagonists (Costa et al. 2004). Similarly, the protective effects against lipopolysaccharides (LPS)-stimulated macrophages induced by CBD occurs via TRPV1, but not CB1 or CB2 receptors, by modulating enhanced Nrf2, toll-like receptor 4 (TLR4) activity, and nuclear factor kappa B (NF-κB) activity (Rajan et al. 2016). Cannabidiol also reduces the increase in inflammatory cytokines and chemokines induced by various inflammatory stimuli via TRPV1 (Etemad et al. 2022; Peyravian et al. 2020) through downstream signalling pathways following the TRPV1 activation including phosphoinositide 3-kinase/protein kinase B (PI3K/Akt) and the extracellular signal-regulated kinase 1/2 (ERK1/2), c-Jun N-terminal kinases/mitogen-activated protein kinase (JNK/MAPK) and the Janus kinase/signal transducer and activator of transcription (JAK/STAT) (Peyravian et al. 2020).

The anticancer effects of CBD can be mediate by both CB2 and TRPV1 receptors. Indeed, it has been reported that CBD can induce apoptosis, modulate cell adhesion, reduce cells viability and promote endoplasmic reticulum (ER) stress as well as oxidative stress (Etemad et al. 2022). Depending on cell types, these effects seem to be mediate by different pathways involving p42/44 MAPK, p38 MAPK, poly ADP ribose polymerase (PARP), transcription factor 4 (ATF4); most of them are related to increase of Ca^2+^ and TRPV1 activity. Moreover, through TRPV1, CBD can modulate autophagy via ERK1/2 activation and Akt but not dependent on the mammalian target of rapamycin complex 1 (mTORC1) (Vrechi et al. 2021).

Cannabidiol has been proposed as the most active pCB for the TRPV2 channel (Etemad et al. 2022). Moreover, the understanding of how CBD affects TRPV2 is hindered by the absence of a specific ligand, and CBD is still employed as an agonist to elucidate the function of TRPV2. In addition to its ability in desensitise the TRPV2 channel, CBD stimulate the synthesis of TRPV2 and its translocation to the plasma membrane. Some antitumoral effects of CBD have been described through TRPV2 and not via other CB receptors (i.e. CB1, CB2, or TRPV1) (Etemad et al. 2022). Cannabidiol resulted able to regulate PI3K/Akt pathway and mTORC1 for promoting cell differentiation and autophagy (Nabissi et al. 2015).

Even if CBD has affinity for TRPV3 and TRPV4, its efficacy was limited at both channels (Etemad et al. 2022).

### Nuclear receptors and gene expression

Phytocannabinoids like eCBs, could act by regulating the gene expression trough some above described signaling pathway, as well as by interacting directly with nuclear receptor/transcription factors including some members of the PPAR family (PPARα, PPARβ/δ, and PPARγ) (Khosropoor et al. 2023). In particular, CBD is an agonist of the PPARγ: it can promote the formation of PPARγ-retinoid X receptor (RXR) heterodimers inducing the transcription of specific genes involved in metabolism (of glucose and lipids), immune response and inflammation. Moreover, CBD increases the PPARγ expression (Hegde et al. 2015; O’Sullivan 2016). Cannabidiol can activate the Nrf2 and inhibits NF-kB signaling also through PPARγ (Khosropoor et al. 2023).

Overall, cannabinoids can act as redox modulators, exerting antioxidant and pro-oxidant effects depending on the dose, time of exposure, and specific cell types (Rybarczyk et al. 2023). Moreover, it has been demonstrated that the antioxidant effects of CBD can occur via TRPV (as reported above) or PPARγ, both of which can promote the nuclear translocation and activation of Nrf2. This transcription factor, by binding to the antioxidant response element (ARE), activates the expression of phase II antioxidant enzymes, including glutathione S-transferase (GST), catalase (CAT), heme oxygenase-1 (HO-1), and manganese-dependent superoxide dismutase (Mn-SOD), thus modulating the cellular antioxidant response (Rybarczyk et al. 2023). Notably, pCBs and specifically CBD and 9Δ-THC exhibited antioxidants proprieties linked to their phenolic groups. They can act as chain-breaking antioxidants like vitamin E (Dawidowicz et al. 2021).

Further, as NF-kB activation depends upon the regulation of cellular redox status, CBD is also considered involved in the crosstalk between NF-kB and Nrf2 (Atalay Ekiner et al. 2022). Cannabinoids are known as downregulators of NF-kB signalling, which has a crucial role in the activation of various pro-inflammatory gene expressions encoding e.g. IL-1β, IL-6, TNF-α, and COX-2 (Atalay Ekiner et al. 2022). The nuclear translocation of active forms (p50, p52, p65, RelB or c-Rel) can occur only after degradation of IkB inhibitors that could be reduced by CBD also via PPARγ (Khosropoor et al. 2023). Cannabidiol seems to prevent NLR family pyrin domain containing 3 (NLRP3)-inflammasome pathway activation by suppressing the expression of key genes like NLRP3 and caspase 1 (Martinez Naya et al. 2023).

### Toll-like receptors

The anti-inflammatory effects of pCBs can be mediated by Toll-like receptors (TLRs) activated by pathogen-associated molecules, acting within the innate immune system (Cui Sun et al. 2024). Indeed, CBD inhibits TLR1-induced IL-1β secretion and TLR2-induced Interferon Gamma-induced Protein 10 (IP-10), IL-1β, IL-6, and TGF-β1 secretion in human monocytes (Sermet et al. 2021). Both THC and CBD also reduce TLR3-induced IP-10 release and IFN-β protein expression, and CBD inhibits TLR4-induced TNFα production in macrophages (Carlisle et al. 2002). Similarly, CBD can impact TLR 5–8 signaling, whereas cannabinoids can also exacerbate TLR-induced inflammatory signaling in a dose-dependent manner and depending on the type of TLR agonism (Cui Sun et al. 2024).

### Opioids and GABA receptors

Finally, pCBs have been proposed as alternative to opioids for pain management (Ang et al. 2023). Indeed, THC and CBD can both also work as allosteric modulators of the opioid receptor µ and δ (Kathmann et al. 2006). In particular, CBD can serve in the control of glutamatergic signalling via N-methyl-D-aspartate (NMDA) receptor-mediated seizures in vivo (Rodríguez-Muñoz et al. 2018). Moreover, CBD can act on GABAergic neurotransmission affecting CNS excitability, probably modulating the composition and arrangement of different subunits that characterise GABA receptors (Ruffolo et al. 2022). On the contrary, Δ(9)-THC does not have any effects on GABA receptors (Lile et al. 2014).

## Localization of cannabinoid receptors

The first studies to define the presence of the ECS were carried out in animal species and it has been found in both vertebrate (mammals, birds, reptiles, and fish) and invertebrates (such as sea urchins, leeches, mussels, nematodes, crustaceans, protozoa, onychophorans) (Della Rocca and Di Salvo 2020; Silver 2019). In non-mammalian species, cannabinoid receptors have been identified in birds (i.e. parrots, zebrafinch, poultry) (Alonso-Ferrero et al. 2006; Stincic and Hyson 2008; Divín et al. 2022), reptiles and fish (i.e. goldfish, zebrafish) (Silver 2019). Notably, it has been demonstrated that the ECS is highly conserved between Danio rerio (zebrafish) and mammals, exhibiting high similarity to that in rodents and human with a parallel CB receptors expression pattern (Lachowicz et al. 2023). Recently, several studies focused on the identification and localization of cannabinoid receptors in various animal species (dogs, cats, horses cattles and pigs) to suggest a therapeutic potential role of *Cannabis* derivatives in veterinary medicine. Table 1 synthetizes the localization of cannabinoid receptors in the different tissues and cells of dogs, cats and horses.

**Table 1:** Localization of cannabinoid receptors in dogs, cats and horses

Localization	Type of receptor	REFERENCES
Dog		
Central nervous system	CB1: claustrum and neocortex, cerebellar cortex, midbrain, medulla oblongata, globus pallidus and substantia nigra, trigeminus, basal ganglia, cochlear nucleus, olfactory bulb and hippocampus.	Pirone et al. 2016 Freundt-Revilla et al. 2017 Silver 2019 Kostic et al. 2023 Chiocchetti et al. 2019
	CB1s decrease in dogs with idiopathic epilepsy and increase in dogs with structural epilepsy.	
Peripheral nerve system	CB1: neurons, myelinating Schwann cells and dorsal root ganglia.	
	CB2: neuron, Schwann cells, blood vessel smooth muscle cells, pericyte-like cells TRPV1: neuron, satellite glial cells	
	GPR55: neuron, satellite glial cells	
	PARRα: satellite glial cells, endothelial cells.	
Immune Cells	CB1 and CB2: peripheral blood mononuclear cells, macrophages.	Brown et al. 2023 Silver 2019
	CB2: mast cells.	
Skin	CB1 and CB2: basal e suprabasal epidermal cell layers, inner epithelial root sheaths cell of hair follicles, reserve cells of sebaceous glands, secretory and ductal cells of sweat glands.	Campora et al. 2012
	CB1 and CB2: perivascular cells with mast cell morphology, fibroblasts, and endothelial cells.	
	CB2: basal e suprabasal cells of outer epithelial root sheaths of hair follicles and cells of arrector pili muscles.	
	Stronger immunoreactivity in skin of dogs with atopic dermatitis.	
	CB1, CB2, GPR55, TRPV1, TRPA1 and PPARα: basal and suprabasal keratinocytes.	Chiocchetti et al. 2022a
	Significant upregulation of CB2 and TRPA1 in samples from dogs with atopic dermatitis.	
	CB2, GPR55, TRPV1 and TRPA1: inflammatory infiltrate of the skin of atopic dogs (mast cells, macrophages/dendritic cells, T lymphocytes).	Chiocchetti et al. 2022b
	GPR55: calprotectin immunoreactive neutrophils.	
	CB1: hair follicles (bulb and suprabulbar region of both primary and secondary hair follicles).	Mercati et al. 2012
Gastrointestinal tract	CB1: enterochromaffin cells of pylorus, small and large intestine; some lamina propria and epithelial cells of small and large intestine.	Galiazzo et al. 2018
	CB2: endothelial and smooth muscle cells of mucosal and submucosal blood vessels, smooth muscle cells of the *muscularis mucosae*, mast cells of the lamina propria, unidentified immunocytes within intestinal lymphatic nodules, muscular layers of the intestine (small intestine > colon > pylorus), neurons and glial cells of intestinal submucosal plexus.	
	GPR55: lamina propria (macrophages, plasma cells and mast cells) and epithelial cells, enterochromaffin cells, muscular layers.	
	PPARα: lamina propria cells, epithelial cells, blood vessels, smooth muscle cells of the *muscularis mucosae* and tunica muscularis, glial cells of submucosal and myenteric plexus.	
Hip and Stifle Joints	CB1, CB2 and GPR55: synoviocytes.	Zamith Cunha et al. 2023a
	CB2 and GPR55: macrophages, neutrophils and vascular cells.	
Mast cell tumors	CB1 and CB2: highly expressed in low-grade mast cell tumors.	Rinaldi et al. 2022
Salivary gland	CB1: epithelial ductal cells.	Dall’Aglio et al. 2010
Embryo	CB1: epithelial immunoreactivity detected in several structures of central and peripheral nervous system, several nervous and non-nervous structures of sensory organs (inner ear structures, the developing eye and the olfactory epithelium), and thyroid.	Pirone et al. 2015
Cat		
Skin	CB1 and CB2: epidermal layers and hair follicle sheaths, differentiated sebocytes and hair bulb matrical cells.	Miragliotta et al. 2018
	CB2: sweat glands.	
	PPAR: basal keratinocytes of epidermis, outer epithelial root sheath and isolated dermal papillae.	
	Overexpression in cats with dermatitis related to hypersensitivity, with the main distribution changes being suprabasal for CB1, dermal for CB2 and marked expression of PPAR-α in hyperplastic epidermis and perivascular infiltrate.	
Oral mucosa	CB1: TRPA1: mucosal epithelium.	Polidoro et al. 2021
	CB2 and GPR55: subepithelial inflammatory cells.	
	Upregulation in cats with chronic gingivostomatitis.	
Gastrointestinal tract	CB1: gastric epithelial cells, intestinal enteroendocrine cells, goblet cells, lamina propria mast cells, and enteric neurons.	Stanzani et al. 2020.
	CB2: intestinal enteroendocrine cells, enterocytes, and macrophages.	
	GPR55: intestinal enteroendocrine cells macrophages, immunocytes, and myenteric plexus neurons.	
	PPARα: immunocytes, smooth muscle cells, and enteroglial cells.	
	TRPA1: enteric neurons and intestinal goblet cells.	
Arterial smooth muscle cells	CB1.	Gebremedhin et al. 1999
Ovary and oviduct	CB1: tertiary follicle granulosa cells, luteal cells, oviduct ciliated cells.	Pirone et al. 2017
Femoropatellar synovial membrane	CB1: in cats with synovitis.	Ruel et al. 2022
Horse		
Metacarpophalangeal synovial membrane	CB1: synoviocytes.	Miagkoff et al. 2023; Zamith Cunha et al. 2023c
	CB2, TRPV1 and PPARα: synoviocytes, blood vessels and fibroblasts.	
	GPR55: synoviocytes and endothelial cells.	
	CB1 and CB2 overexpressed in presence of synovitis; CB1 decreased in horses with osteoarthritis.	
Ileum	CB1: enterocytes, enteric neurons and enteric glial cells of the myenteric and submucosal plexus.	Galiazzo et al. 2021
	CB2: enterocytes, subclasses of immune cells in the lamina propria.	
	PPARα: peripheral smooth muscle cells of the longitudinal muscular layer, neurons and glial cells.	
	TRPA1: neurons of the myenteric and submucosal plexus and the nerve fibers.	
Skin	CB1 and CB2: epidermis, dermal fibroblast-like cells, sebaceous glands, sweat glands.	Kupczyk et al. 2022;
	CB2: hair follicles, endothelial cells and their perivascular cells (pericytes, vascular smooth muscle).	
Trigeminal ganglion	CB1, CB2, TRPV1, GPR55 and PARRγ.	Zamith Cunha et al. 2023b
Dorsal root ganglia	CB1, CB2, PPARα and γ, TRPV1, TRPA1, GPR3 and GPR55.	Chiocchetti et al. 2021 Galiazzo et al. 2022

CB1: cannabinoid receptor type 1; CB2: cannabinoid receptor type 2; GPR55 and GPR3: G protein-coupled receptor 55 and 3; PPAR: peroxisome proliferator-activated receptor; TRPV1: transient receptor potential vanilloid 1; TRPA1: transient receptors potential ankyrin 1

### Localization of CB receptors in dogs

Dogs express a large number of CB1 receptors in the brain, specifically in clastrum and neocortex, cerebellar cortex, midbrain, medulla oblongata, gray matter of spinal cord, trigeminus, basal ganglia, cochlear nucleus, olfactory bulb and hippocampus (Pirone et al. 2016; Freundt-Revilla et al. 2017; Silver 2019). In the hippocampus, a different expression of CB1 receptors was observed in dogs affected by idiopathic or structural epilepsy when compared with healthy subjects. Specifically, a significant decrease of CB1 receptors was observed in dogs with idiopathic epilepsy, while an increase of the same receptors was reported in dogs with structural epilepsy (Kostic et al. 2023). At the dorsal root ganglia level, besides CB1 receptors, also TRPV1, GPR55 and CB2 receptors were identified. In satellite glial cells, a strong presence of CB2 receptors but also of PPARα and TPVR1 was observed in old dogs. A clear presence of CB2 receptors was observed in Schwann cells and cells surrounding blood capillaries and, although at a lesser extent, in smooth muscular blood vessels (Chiocchetti et al. 2019). The distribution and density of cannabinoid receptors show interspecies differences. In dogs, CB1 receptors are significantly more expressed in hind brain than in humans (Silver 2019).

At the gastrointestinal tract level, a large presence of cannabinoid receptors, both CB1 and CB2 and also GPR55 and PPARα was observed. Specifically, immunoreactivity for cannabinoid receptors was found in the lamina propria (CB1, CB2 and GPR55), epithelial cells (CB1), mast cells (CB2), immunocytes (CB2), blood vessels (CB2 and PPARα), smooth muscle cells (CB2, GPR55 and PPARα), macrophages (GPR55), neurons and glial cells of the submucosal plexus (CB2) and glial cells of the myenteric plexus (PPARα) (Galiazzo et al. 2018).

Cannabinoid receptors CB1 and CB2 are also present in skin of healthy dogs and dogs with atopic dermatitis, with a stronger immunoreactivity in diseased subjects (Campora et al. 2012). More recently, also GPR55, TPVR1, PPARα and ankyrin 1 (TRPA1), besides CB1 and CB2 receptors, were found in keratinocytes of healthy and atopic dogs, and an overexpression of CB2 receptor and TPRA1 was observed in those with atopic dermatitis (Chiocchetti et al. 2022a). Receptors such CB2, GPR55, TPVR1, PPARα and TRPA1 were also found in inflammatory infiltrate of the skin of atopic dogs (Chiocchetti et al. 2022b).

Other location of canine CB1 receptor include salivary glands as parotid and mandibular glands (Dall’Aglio et al. 2010), hair follicles (Mercati et al. 2012), and embryo (Pirone et al. 2015).

Both CB1 and CB2 receptors were found in canine mast cell tumors (Rinaldi et al. 2022), immune cells (Brown et al. 2023) and synovial cells of canine hip and stifle (Zamith Cunha et al. 2023a). Synoviocytes express immunoreactivity also for GPR55 (Zamith Cunha et al. 2023a).

### Localization of CB receptors in cats

Similarly to dogs, also in cats PPARα, CB1 and CB2 receptors were overexpressed in the skin of subjects with dermatitis due to hypersensitivity compared to healthy ones (Miragliotta et al. 2018).

A role of ECS was also hypothesized in cats with feline chronic gingivostomatitis: CB1, CB2, GPR55 and TRPA1 were markedly upregulated in the oral mucosa of affected cats when compared to healthy ones (Polidoro et al. 2021). As in dogs, cannabinoid (CB1 and 2) and cannabinoid-related receptors (GPR55, PPARα and TRPA1) are widely expressed in the gastrointestinal tract of cats (Stanzani et al. 2020).

Lastly, CB1 receptors are also expressed in feline arterial smooth muscle cells (Gebremedhin et al. 1999), ovary and oviduct (Pirone et al. 2017) and in synovial membrane of cats with synovitis (Ruel et al. 2022).

### Localization of CB receptors in horses

In horses, the most recent scientific literature has focused on the identification of cannabinoid receptors in the synovial membrane of metacarpophalangeal joint. CB1 and CB2 receptors, as well as TRPV1, GPR55 and PPARα, were found in synovia of healthy horses (Miagkoff et al. 2023; Zamith Cunha et al. 2023c). Miagkoff et al. (2023) found a relationship between the expression of CB1 and CB2 receptors in the synovia and the grade of synovitis or osteoarthritis in equine metacarpophalangeal joints. They observed that CB1 and CB2 were overexpressed with increased degree of synovitis, while CB1 receptors significantly decreased with the increase of osteoarthritis scores (Miagkoff et al. 2023).

Cannabinoid receptors were also widely distributed in ileum (CB1 and CB2 receptors, PPARα and TRPA1), skin (CB1 and CB2 receptors), in the trigeminal ganglion (CB1, CB2, TRPV1, GPR55 and PARRγ) and dorsal root ganglia (CB1 and CB2, PPARα and γ, TRPV1, TRPA1, GPR3 and GPR55) (Chiocchetti et al. 2021; Galiazzo et al. 2021, 2022; Kupczyk et al. 2022; Zamith Cunha et al. 2023b).

### Localization of CB receptors in other animal species

The information available regarding other animal species is very scarce. CB2 receptors were found in bovine fetal pancreas (Dall’Aglio et al. 2017), while CB1 receptor was identified in pig clastrum (Pirone et al. 2020) and myenteric plexus of pig ileum together to TRPV1 and TRPA1 (Toschi et al. 2021). Moreover, the expression of genes encoding CB1 receptor in spermatozoa of bull was correlated to animal fertility: a major expression of this gene was found in sample from high fertile bulls compared to low fertile ones (Kumar et al. 2018).

## Pharmacokinetics of phytocannabinoids in animal species

The understanding of the pharmacokinetics (PK) of a drug is crucial for defining an accurate dosage regimen and avoiding its empirical use. Consequently, the increasing interest in the therapeutic application of pCBs in veterinary clinical practice has led to a growing number of publications on their pharmacokinetics in various animal species in recent years. Studies aimed at defining the kinetic behavior of cannabinoids primarily focus on CBD, especially following oral administration in dogs (Bartner et al. 2018; Gamble et al. 2018; Deabold et al. 2019; Chicoine et al. 2020; Fernández-Trapero et al. 2020; Wakshlag et al. 2020; Kulpa et al. 2021; Vaughn et al. 2021; Doran et al. 2022; Polidoro et al. 2022; Tittle et al. 2022; Corsato Alvarenga et al. 2023; Della Rocca et al. 2023; Limsuwan et al. 2024). More recently, similar studies have been conducted in horses (Ryan et al. 2021; Turner et al. 2022; Williams et al. 2022; Yocom et al. 2022; Eichler et al. 2023a, 2023b; Sánchez De Medina et al. 2023; Thomson et al. 2024) and cats (Deabold et al. 2019; Kulpa et al. 2021; Wang et al. 2022; Jukier et al. 2023; Rozental et al. 2023a; Lyons et al. 2024). However, as of now, the pharmacokinetic profile of CBD has not been fully elucidated in any of these species.

### Absorption and bioavailability

According to the authors’ knowledge, only a few experimental studies, one in dogs and two in horses, have administered CBD both orally and intravenously, allowing for the estimation of its oral bioavailability. The results indicated a bioavailability of less than 19% in dogs (Samara et al. 1988) and 14% in horses (Sánchez De Medina et al. 2023; Turner et al. 2022). A poor bioavailability may correspond to a lack of therapeutic efficacy, and from a pharmacokinetic perspective, a significant interindividual variability is more likely to be observed (Millar et al. 2020).

A considerable variability in CBD plasma concentrations following oral administration was observed in the mentioned studies in dogs, horses, cats, but also in rabbits (Rooney et al. 2022), calves (Meyer et al. 2022), and parrots (Sosa-Higareda et al. 2023). The reduced oral bioavailability of CBD has been attributed to both poor intestinal absorption and high hepatic metabolism (Perucca and Bialer 2020). Various formulations, such as microencapsulated CBD oil beads, CBD-infused oil (Bartner et al. 2018), nanoemulsion, water-soluble and semisolid forms (Limsuwan et al. 2024), CBD-rich hemp extract in soft gel capsules and sesame oil (Tittle et al. 2022), soft chews containing a CBD/CBDA predominant extract, and the same extract diluted in different oils (Wakshlag et al. 2020), were tested. Different routes of administration that can avoid first-pass metabolism, as sublingual (Fernández-Trapero et al. 2020), transdermal (Bartner et al. 2018), intranasal, intrarectal (Polidoro et al. 2022), and oral transmucosal (Della Rocca et al. 2023), were evaluated, but unfortunately, conclusive results were not achieved. It is important to note that the diverse experimental conditions used in various studies, such as sampling times, the number of samples, the number of animals enrolled, age, sex, and breed, make comparisons among them very difficult.

After multiple oral escalation doses of CBD (up to a maximum dose of 30.5 mg/kg) in cats, the observed maximum concentration (Cmax) was about half that obtained with co-administration of a THC/CBD oil orally administered in escalating doses up to a maximum of 10.6 mg/kg of CBD (Kulpa et al. 2021). This result led to the hypothesis that THC may improve CBD absorption (Rozental et al. 2023a). More recently, an increase in the oral bioavailability of CBD in rats was observed following oral administration of CBD as a full spectrum compared to an equal dose of CBD alone. In the same study, the authors also demonstrated, through in vitro assays, that THC can increase the intestinal permeability of CBD and decrease its efflux (Berthold et al. 2023).

Previously, a greater Cmax and Area Under Curve (AUC) have already been observed for CBDA following oral administration of a full spectrum extract (CBDA in co-presence of other cannabinoids) when compared with the administration of pure CBDA in mice (Anderson et al. 2021). Regarding this issue, the different types of cannabinoids (full spectrum, purified, or synthetic) used in various pharmacokinetic studies introduce another variable in elucidating their kinetic behavior.

In humans, it was observed that a high-fat meal enhances the absorption of CBD, allowing for Cmax and AUC values about four times higher than those resulting from a fasted state (Taylor et al. 2018). Similar results have been reported in cats, where a significantly higher AUC was observed for orally administered CBD 30 min after a meal compared to its administration in the fasting state (Jukier et al. 2023). In dogs, the data obtained are not entirely conclusive; even if the Cmax value of CBD was significantly higher under fed conditions compared with a fasted state, no significant difference was observed for AUC values (Doran et al. 2022). It is essential to underline that, in this last study, only three subjects per group were enrolled, and for one dog, the greater values of Cmax and AUC were obtained in the fasted condition. On the contrary, following oral administration of an oil containing CBD and CBDA in rabbits, the AUC values of both cannabinoids were significantly greater in the fasted state than after a slurry meal (Rooney et al. 2022). Also, for THC, CBG, and CBGA, a greater extent of oral absorption, with higher values for both Cmax and AUC, was observed in fasted dogs compared to fed dogs (Łebkowska-Wieruszewska et al. 2019; Amstutz et al. 2022).

Contrarily to single administration, after oral treatment with a CBG and CBGA-rich hemp extract twice a day for two weeks in fed and fasted dogs, no significant differences in blood concentrations of cannabinoids were observed between the two different food states (Amstutz et al. 2022). Changes in the extent of absorption can be due to species-specific differences and/or depend on the nature and amount of ingested food (Deng et al. 2017). The variables linked to food can sometimes also influence the rate of absorption, as observed following the administration of THC in fed dogs where time to maximum concentration (Tmax) resulted in 5 h compared to 1.25 h observed in fasted subjects (Łebkowska-Wieruszewska et al. 2019).

Additionally, the acid forms are generally more absorbed than the corresponding non-acid products. This phenomenon was observed after single oral co-administration of CBD and CBDA in similar concentrations in dogs (Wakshlag et al. 2020; Tittle et al. 2022), cats (Wang et al. 2022), rabbits (Rooney et al. 2022), horses (Thomson et al. 2024) and parrots (Sosa-Higareda et al. 2023), as well as following transdermal application, twice a day for two weeks, in dogs (Hannon et al. 2020). In all cases, both Cmax and AUC values of CBDA were at least double that of CBD. This was also observed for other acid derivatives, such as THCA vs. THC in dogs, cats and horses (Wakshlag et al. 2020; Tittle et al. 2022; Wang et al. 2022; Thomson et al. 2024) and CBGA vs. CBG in cats and dogs (Amstutz et al. 2022; Wang et al. 2022). It should be highlighted that, after multiple oral administrations, the differences in absorption of CBD and CBDA, observed after a single administration, were attenuated and similar in both dogs and parrots (Wakshlag et al. 2020; Sosa-Higareda et al. 2023).

Following oral administration of industrial hemp (flower material in gelatine capsules) in cattle, the average Cmax of THCA-A, CBCA, and CBDVA was 12.1, 12.3, and 13.1 ng/mL, respectively. Considering that cattle were exposed to very different doses of these cannabinoids (117.4 mg of THCA-A, 101.3 mg of CBCA, and 3.5 mg of CBDVA), it is possible to suppose that CBDVA has the highest oral bioavailability from cattle rumen compared to the other acid cannabinoids (Kleinhenz et al. 2020).

### Distribution

Following intravenous administration of THC in swine and CBD in horses, a large apparent volume of distribution was computed, indicating a deep distribution of these cannabinoids in the organism (Schaefer et al. 2016; Sánchez De Medina et al. 2023). In swine, intravenously administered THC was primarily distributed in bile fluid, lung, and perirenal and abdominal adipose tissue. However, it was also detected in the brain, heart, liver, kidney, spleen, and muscle (Schaefer et al. 2017). In sheep, after being fed for 56 days with diets containing different percentages of hemp stubble, with THC content less than or equal to 0.001% on a dry matter basis, the cannabinoid was detected in kidney fat, subcutaneous fat, and loin meat, although not in all animals (Krebs et al. 2021). In sheep, when fed for 22 days with a pellet diet containing 42% green hemp (partly in full flower), THC was detected in the subcutaneous fat of 4 out of 6 animals at the end of the treatment, and 35 days later it was found in all animals, albeit at lower concentrations. These results led the authors of the study to hypothesize the existence of a redistribution of THC from other body compartments into fat (Stevens et al. 2022). Further, cannabinoid can accumulate in milk; following seven days of feeding with hemp silage, which had low cannabinoid concentrations, cow’s milk showed detectable levels of Δ9-THC, Δ9-THCA, Δ9-THCV, CBD, CBN, and CBDV. After an additional six days with a higher cannabinoid content in the diet, the milk concentrations of Δ9-THC, Δ9-THCV, and CBD were higher than plasma concentrations by over 6, 3, and 11 times, respectively (Wagner et al. 2022).

Finally, CBD was detected in the synovial fluid of 5 out of 6 horses following a single oral administration of 3 mg/kg of CBD in sunflower lecithin oil. Furthermore, its synovial concentrations consistently increased at five weeks after oral administration of the same formulation at 1.5 mg/kg twice daily, indicating a probable cumulative effect in this compartment (Yocom et al. 2022).

Although higher blood concentrations were observed for acidic cannabinoid derivatives than their non-acidic counterparts following the administration of equal doses, they do not appear to have the same ability to cross the blood-brain barrier. Indeed, the brain/plasma AUC ratios of CBD and CBDA following intraperitoneal administration in mice resulted in 0.51 (Deiana et al. 2012) and 0.04 (Anderson et al. 2019), respectively. Tiny brain/plasma AUC ratios, between 0.02 and 0.04, were also observed for other acid derivatives (CBDVA, CBGVA, and CBGA), while for CBCA and THCA, their presence at the central nervous system level was not detectable at all (Anderson et al. 2019).

### Metabolism

In terms of cannabinoid metabolism, as lipophilic molecules, pCBs undergo extensive biotransformation reactions. In humans, CBD is primarily converted into the active metabolite 7-hydroxy cannabidiol (7-OH-CBD) and is further metabolized into 7-carboxy cannabidiol (7-COOH-CBD), which is the most abundant circulating metabolite; 6α- and 6β-hydroxy cannabidiol (6-OH-CBD) are also formed but in smaller amounts (Mechoulam and Hanuš 2002; Pérez-Acevedo et al. 2020; Sitovs et al. 2024). Regarding hydroxylated metabolites, 6-OH-CBD was the major metabolite in dogs following the oral administration of a cannabis herbal extract. 7-OH-CBD was only intermittently observed (Chicoine et al. 2020). Similarly, in cats treated with the same formulation at the same doses, only 6-OH-CBD was detected, albeit with blood concentrations 2.5 fold smaller than in dogs (Lyons et al. 2024), indicating species differences in biotransformation. In humans, 7-OH-CBD is predominantly formed via CYP2C19 and CYP2C9 isozymes, while in a canine in vitro study, the main enzyme of the CYP2C subfamily (CYP2C21) is of secondary importance in CBD metabolism compared to CYP1A2. In humans, CYP1A2 appears to be less involved in CBD metabolism and does not form the active metabolite 7-OH-CBD (Court et al. 2024).

Similar to what is observed in humans, a greater presence of 7-COOH-CBD compared to the hydroxylated metabolites was reported in the serum of dogs (Chicoine et al. 2020; Wakshlag et al. 2020; Vaughn et al. 2021), cats (Wang et al. 2022), and horses (Ryan et al. 2021; Turner et al. 2022; Eichler et al. 2023a, 2023b). In cats, after oral administration of 11 escalating doses of both CBD and CBD/THC combined oil, the concentration of the metabolite 7-COOH-CBD at 24 h post-dosing was significantly higher following treatment with combined oil, even if the total dose of CBD administered was smaller (Kulpa et al. 2021). Conversely, in parrots, following a single oral administration of 30/32 mg/kg of CBD/CBDA-rich hemp extract, this metabolite was not quantifiable in serum (Sosa-Higareda et al. 2023). In this last study, the predominant metabolite observed was 11-OH-THC, due to a concentration of THC and THCA in the formulation such that the administered dose was 1.23 and 0.39 mg/kg, respectively. These results indicate another species-specific difference in cannabinoid biotransformation (Sosa-Higareda et al. 2023).

THC is another cannabinoid that undergoes extensive metabolism, and the main biotransformation products observed in humans are 11- hydroxy tetrahydrocannabinol (11-OH-THC) and 11- carboxy tetrahydrocannabinol (11-COOH-THC) (Lucas et al. 2018). In dogs, following single sublingual administration of Sativex (dose equal to 8.1 mg of THC and 7.5 mg of CBD), the hydroxylated metabolite, 11-OH-THC, was observed, albeit at the limit of detection; its Cmax and AUC increased following two weeks of treatment. 11-COOH-THC was not tested for its presence in serum (Fernández-Trapero et al. 2020). Sporadically, 11-OH-THC was observed in canine serum after oral administration of CBD/CBDA-rich hemp extract in different oils (Wakshlag et al. 2020) and Cannabis herbal extract (Chicoine et al. 2020); the latter study is the only one to report, albeit only for some samples, the detectable presence of 11-COOH-THC in dogs. The formation of 11-OH-THC was also observed in cats following oral administration of 11 escalating doses of both THC alone and CBD/THC combined oils. Surprisingly, 4 h after the end of administrations, a significantly higher peak concentration was observed following CBD/THC combined oil, even if the maximum THC dose administered with this formulation was 8.4 mg/kg vs. 41.5 mg/kg of the THC-alone oil (Kulpa et al. 2021). In a more recent study neither 11-OH-THC and 11-COOH-THC were detected in blood of cats orally treated with 1:20 THC: CBD cannabis herbal extract at the dose of 0.25 mg/kg in THC (Lyons et al. 2024).

### Elimination

Cannabidiol and its metabolites are excreted in feces and urine (Bradley et al. 2022; Ryan et al. 2021; Samara et al. 1990). The presence of different metabolites in dogs’ urine confirms the existence of its extensive metabolism in this species (Samara et al. 1990). Both 7-OH-CBD and 7-COOH-CBD were detected in equine urine up to 72 h after oral administration of CBD. However, it is necessary to underline that urine samples were exposed to the β-glucuronidase enzyme, so the two detected metabolites also account for any conjugate derivatives (Ryan et al. 2021). Cannabidiol, as such, was also found in canine urine, although in lower amounts than observed in feces (Bradley et al. 2022). In the opinion of the authors of this review, however, it cannot be ruled out that the higher concentrations of CBD in feces are related to its unabsorbed portion rather than its biliary elimination.

## Efficacy and tolerability of phytocannabinoids in dogs, cats, horses and cattle

### Dogs

Most studies aimed at evaluating the efficacy and tolerability of *Cannabis* derivatives in veterinary medicine are mainly focused on CBD in the canine species. Pathologies considered include pain (especially from osteoarthritis), epilepsy, behavioral and skin disorders.

The efficacy and safety of cannabis derivatives in treating chronic pain have been tested in ten studies, while only two studies dealt with the use of CBD in acute painful conditions.

From the randomized, placebo-controlled, double-blind, crossover clinical trials conducted by Gamble et al. (2018), Brioschi et al. (2020), and Verrico et al. (2020) to evaluate the therapeutic effects of different *Cannabis* formulation containing mainly CBD in dogs with chronic osteoarthritic (OA) pain, CBD seemed able to significantly reduce pain and increase locomotor activity, thus improving dogs’ quality of life (Gamble et al. 2018; Brioschi et al. 2020; Verrico et al. 2020). However, these results are not in line with those obtained by Mejia et al. (2021) in their double-blind, randomized, placebo-controlled, crossover clinical study conducted on OA patients, where no differences were noted between groups for any of the recorded outcome measures (Mejia et al. 2021). In addition to the above studies, two pilot studies (Martello et al. 2019; Shilo-Benjamini et al. 2023), a non-blinded observational study (Kogan et al., 2020) and two case reports (Coelho et al. 2021; Shilo-Benjamini et al. 2022), all conducted in OA dogs, have also reported the positive therapeutic effects of CBD administration on pain and locomotion. Very recently, a double-blind, placebo-controlled study was also performed in owned dogs with chronic pain (the origin of which was not specified). The study was a 16-week evaluation of dogs in either placebo or hemp oil phase, each lasting 8 weeks. Results showed a decrease in pain scores as well as in plasma levels of proinflammatory cytokines. However, the lack of washout between the phases was a procedural limitation of this study. More detailed information on these cited studies is reported in Table 2.

**Table 2:** Summary of studies on the efficacy of *Cannabis* derivatives in dogs

Study design, formulation, dose, treatment duration and *n*. of recruited dogs	Efficacy	Tolerability	Reference
Cannabis derivatives in chronic pain from osteoarthritis (OA)			
Randomized, placebo-controlled, double-blind, crossover clinical trial to evaluate the analgesic efficacy of a CBD-dominant hemp oil (equal mix of CBD and CBDA, 2 mg/kg orally twice daily for four weeks) on OA-related pain relief in 16 dogs.	CBD produced a significant decrease in pain scores measured by the Canine Brief Pain Inventory (CBPI) and increased activity levels measured by the Hudson activity scale at weeks 2 and 4 during CBD treatment compared to baseline (week 0) and placebo.	Owners reported no side effects; however, serum chemistry showed an increase in alkaline phosphatase (ALP) activity during CBD treatment in 9 out of 16 treated dogs.	Gamble et al. 2018
Randomized, placebo-controlled, double-blind clinical trial to evaluate the analgesic potential of different doses and formulations of hemp-derived CBD oil on OA-related pain in 20 dogs. Dogs received 20 or 50 mg/day of naked CBD, or 20 mg/day of liposomal CBD, or placebo, orally, for four weeks.	CBD significantly increased mobility and reduced pain scores measured by the Helsinki Chronic Pain Index (HCPI) in a dose-dependent manner, with liposomal CBD being as effective as the higher dose of non-liposomal CBD in improving clinical outcomes.	No relevant changes in cell blood counts and biochemical profile were reported following treatments.	Verrico et al. 2020
Randomized placebo-controlled study to evaluate the efficacy of a pure CBD oil formulation in relieving OA-related pain in 9 dogs. CBD was given by oral transmucosal route (OTM, 2 mg/kg CBD twice daily for 12 weeks), within a multimodal analgesic drug regimen.	The OTM CBD improved owner-reported pain scores and quality of life of CBD treated dogs as measured by the CBPI.	Minimal ptyalism, somnolence and mild ataxia were recorded in 2 and 3 dogs in the CBD-treated group, respectively. No relevant changes in the blood cell count and serum biochemical analysis were noted.	Brioschi et al. 2020
Double-blind, randomized, placebo-controlled, crossover clinical study to evaluate the efficacy of a CBD-dominant hemp oil (2.5 mg/kg of CBD orally twice daily for six weeks) on OA-related pain in 23 dogs.	No differences were observed between groups at any time point for any of the outcomes (objective gait analysis, activity counts and pain scores measured by the Liverpool Osteoarthritis in Dogs– LOAD– and the CBPI).	Vomiting in 1/24 dogs, and mild elevation in liver enzymes in 14/24 were recorded.	Mejia et al. 2021
Pilot clinical study to evaluate the efficacy of a dietary supplement containing CBD, *Boswellia serrata* and *Cucumis melo* on OA-related pain in 8 dogs. The daily quantity of CBD was about 2.4 mg/15 kg BW for 30 days.	A significant reduction in pain scores measured by the HCPI at the end of the study was recorded.	No adverse effects were observed.	Martello et al. 2019
Pilot clinical study to evaluate the therapeutic efficacy of a single subcutaneous injection of liposomal-CBD (5 mg/kg) on OA-related pain in 6 dogs. Dogs were monitored for six weeks after treatment.	Dogs showed significantly improved CBPI pain severity scores compared with baseline at weeks 2 and 3, improved CBPI pain interference scores at weeks 2 and 6, improved CBPI total scores at weeks 2 and 3 and borderline improvement at week 6, and improved CBPI quality of life at weeks 2 and 3. Collar activity scores were significantly increased on weeks 5 and 6.	The main adverse effect was minor local swelling for several days in 5/6 dogs.	Shilo-Benjamini et al. 2023
Non-blinded observational study to evaluate the efficacy of a CBD-dominant full-spectrum hemp oil-based product (0.3–4.12 mg/kg of CBD orally twice daily for 90 days) as adjunctive therapy (dogs were under multimodal analgesic therapy - acupuncture, laser, nutraceuticals, polysulfated glycosaminoglycan, and/or gabapentin) on OA-related pain in 32 dogs.	Thirty out of 32 dogs showed pain relief (as measured on a 0 to 10 scale, with 0 representing no pain and 10 the worst possible pain), and 21/23 dogs could reduce or discontinue gabapentin.	An increase in ALP activity was the only detected adverse effect.	Kogan et al., 2020
Case report on one dog with chronic OA treated with a CBD-purified hemp oil (1 mg/kg orally twice daily for 30 days).	CBD treatment produced analgesia with consequent improvement in mobility and quality of life of the dog.	N.R.	Coelho et al. 2021
Case report on a dog subcutaneously injected with a liposomal cannabidiol formulation (5 mg/kg) for pain management as compassion care. The dog was cachectic, presented general muscle atrophy, suffered from bilateral hip and elbow osteoarthritis and severe cervical pain, and had a suspected testicular neoplasia. Notwithstanding a multimodal analgesic treatment on board, the dog deteriorated rapidly, with increased pain, and decreased function.	After the CBD injection, the dog showed improved CBPI and Interactive Visual Analog Scale (iVAS) pain scores, and increased collar activity scores (compared to his activity before the injection) up to 3 weeks following injection.	N.R.	Shilo-Benjamini et al. 2022
*Cannabis* derivatives in chronic pain (origin not detailed)			
Double-blind placebo-controlled study to evaluate the efficacy of a hemp oil (containing 15 mg/ml total cannabinoids) in organic extra virgin olive oil (2 mg phytocannabinoids/kg orally twice a day for 8 weeks, and placebo for the previous or the following 8 weeks, depending on group allocation of dogs) in 40 dogs living with pain.	The intervention was positively associated with a decrease in pain scores (based on CBPI), increased ability to walk up and down the stairs, and improved daily activity. Decreases in plasma levels of proinflammatory cytokines TNF-α, IL-6, and IL-8 were also observed.	No major adverse effects attributable to the intervention were reported during the study. The incidence of vomiting or diarrhea were rare and equally distributed between the intervention and placebo groups. Blood work showed no significant changes in most of the parameters tested.	Panda et al. 2024
*Cannabis* derivatives in epilepsy			
Randomized placebo-controlled, double-blinded clinical trial to assess the efficacy of a CBD-infused hemp oil (2.5 mg/kg orally twice daily for 12 weeks) in addition to conventional antiepileptic treatment on seizure frequency in 26 dogs with idiopathic epilepsy.	Compared to the placebo group, the CBD-treated group exhibited a significant reduction in seizure frequency (median change, 33%). However, there were no differences between groups in the proportion of dogs considered responders to treatment (≥ 50% decrease in seizure activity).	The only adverse effect was increased ALP activity.	McGrath et al. 2019
Randomized, controlled-placebo, crossover study to evaluate the efficacy of a CBD and CBDA-rich hemp product (2 mg/kg orally twice daily for 12 weeks) for the treatment of refractory epileptic seizures in 14 dogs.	The CBD treatment produced a statistically significant reduction in epileptic seizure frequency and number of epileptic seizure days compared to the placebo group.	Mild adverse events included vomit/diarrhea, somnolence, anxiety, and mild worsening of ataxia, occurring in 2, 3, 2 and 4 treated subjects, respectively.	Garcia et al. 2022
Double-blinded placebo-controlled crossover study to evaluate the efficacy of a CBD-infused hemp seed oil on total seizures and seizure days in 51 dogs with at least two seizures per month while receiving at least one antiseizure drug (ASD), with phenobarbital, potassium bromide, zonisamide and levetiracetam being the most used ASD. As the initial tested 5 mg/kg/day dose did not produce any change in 12 dogs, a dosage of 9 mg/kg/day (orally for three months) was used in the following 39 dogs.	At the lower CBD dose, no significant changes to total seizures or seizure days were observed. At the higher dose, a slight increase in the percentage change (3.31%) of total seizures from baseline was recorded, which was significantly lower compared to the placebo group (30.72%). Conversely, the percentage change of seizure days in the CBD group decreased significantly by 24.1%, whereas dogs on placebo had a 5.81% increase. A percentage change ≥ 50% (responders) was observed in 9 subjects in the CBD group and 8 in the placebo group for total seizures, and in 13 versus 8 subjects for seizure days. These differences were not significant.	Significant increase in mean serum ALP and alanine aminotransferase (ALT) activity in the CBD group. Owners reported various adverse effects, including decreased or increased appetite, vomiting, soft feces or diarrhea, anxiety, increased or decreased activity, ataxia, and aggression. Among these, decreased appetite and vomiting were more frequently reported in the CBD group.	Rozental et al. 2023b
Case series to evaluate the efficacy of a CBD-predominant full-spectrum hemp oil in 3 dogs with suspected epilepsy. Dogs were treated respectively with different doses of CBD (0.51 mg/kg, 1.24–1.25 mg/kg and 5 mg/kg, respectively), given orally twice daily for eight weeks.	Results varied among dogs, with one experiencing a considerable reduction in epileptic seizure frequency and improvement of other signs (i.e., aggression behavior), another showing slight improvement of seizure intensity, and the third showing no response to therapy, as reported by the owners.	Somnolence in 2/3 dogs was the only reported adverse effect.	Mogi and Fukuyama 2019
*Cannabis* derivatives in behavioral disorders			
Replicated 4 × 4 Latin square design experiment to evaluate the influence of a CBD industrial hemp extract incorporated into treats on behavioral responses (such as cowering, shaking, vocalization, destructiveness, and tucking tail upon the start of the fireworks track) to fear-inducing stimuli in 16 dogs. CBD was dosed at 1.4 mg/kg orally 4–6 h prior to the test.	The obtained results did not provide strong support for the anxiolytic effect of CBD in dogs.	N.R.	Morris et al. 2020
Placebo-controlled study to determine if a 5% CBD-based oil (dosed at *~* 1.25 mg/kg orally once a day for 45 days) could affect stress related behavior in 12 shelter dogs.	Aggressive behaviour towards humans decreased significantly over time in the CBD treatment group. However, only the T0-T2 (baseline - 45th day) comparison was significant in the pairwise comparisons.	One-day duration diarrhea (1/24) was the only reported side effect.	Corsetti et al. 2021
Blinded, placebo-controlled, parallel-design study to determine the anxiolytic effect of a CBD based hemp derived distillate incorporated into soft gel capsules (~ 4 mg/kg of CBD orally 2 h prior to the test) in dogs experiencing a separation event (*n*=21) or a car travel (*n*=19).	The mitigating effect of CBD treatment varied by outcome. measures and tests, with some indicating a significant reduction in canine stress compared to the placebo group.	N.R.	Hunt et al. 2023
Blinded, parallel study design to determine whether multiple doses of a tetrahydrocannabinol-free CBD distillate (4 mg/kg of CBD orally 2 h prior the tests) over a period of 6 months could positively influence measures of stress due to a series of short car journeys (test) in 19 dogs.	The mitigating effect of CBD treatment varied by measure, with cortisol, whining, lip licking, and qualitative behavioral ratings indicating a significant reduction in canine stress compared to the placebo group for at least one time point. The effect of CBD decreased over time following 6 months of daily treatment.	N.R.	Flint et al. 2024
Pilot study to evaluate the effects of CBD administration (2.0 mg/kg/day orally over a 2-week-period) compared to placebo on the vocal activity of 10 healthy domestic dogs upon their temporary separation from caregivers.	All dogs vocalized more often when being left alone, regardless they had received CBD or placebo, but the degree of such increase was significantly less robust following the CBD administration, probably due to an anxiolytic effect of CBD.	N.R.	Masataka 2024
*Cannabis* derivatives in skin diseases			
Randomised, double-blinded and placebo-controlled trial to determine if the administration of gelatin capsules containing a CBD/CBDA-rich hemp extract (2 mg/kg orally twice daily for 28 days) as an adjunct therapy decreased pruritus and cutaneous lesions in 17 dogs with atopic dermatitis.	Lesion severity was not affected by CBD/CBDA; however, the treatment did have a positive effect on pruritus, as scored with the Pruritus Visual Analog Scale (PVAS, a 0 to10 scale, with 0 representing a complete absence of pruritus and 10 the most severe form of pruritus), in some dogs.)	Adverse effects were lethargy (2/17), somnolence and sleepiness (2/17), decreased aggression (1/17) and increased calmness (3/17), regurgitation (1/17), increased flatulence (1/17), loss of appetite (1/17), increased energy/mobility (2/17). While not significant, ALP was elevated outside the reference range in six of 17 treatment group dogs on the 28^th^ day.	Loewinger et al. 2022
Double-blinded and placebo-controlled trial to evaluate the effectiveness of a full-spectrum cannabis oil rich in CBD (2.5 mg/kg orally twice daily for 60 days) in 14 dogs with atopic dermatitis.	No differences regarding lesions’ severity and pruritus in pre- and post-treatment were obtained.	N.R.	Mariga et al. 2023
Retrospective case series examining the effect of a 10% CBD-containing broad-spectrum hemp oil as a supplemental treatment for canine atopic dermatitis in 8 dogs. CBD treatment started with an initial dose of approximately 0.07 to 0.25 mg/kg orally twice daily; then, the dose was increased up to 0.72 mg/kg if no apparent change was observed with the previous dose. Administration was performed for at least eight weeks.	While there was relatively little change in lesion severity, CBD decreased the occurrence of pruritus, as scored with a PVAS, in 6/8 dogs.	No adverse events were reported following the ingestion of the CBD oil.	Mogi et al. 2022
Case report of a dog with discoid lupus erythematosus (DLE) resistant to conventional treatment. The dog was treated orally as follow: initial dose 1 drop/day (0.08 mg/kg) of a full-spectrum oil containing a 2:1 THC: CBD ratio (40 mg/ml); the dose was then modified (increased and decreased), up to a final dose of 3 drops once daily (0.24 mg/kg total cannabinoids) and the adjunct of 10 drops of a full-spectrum CBD-rich oil (50 mg/mL) twice daily (1.96 mg/kg/day total cannabinoids).	Significant improvement in skin lesions within a few weeks was observed, and after 1 year the dog remained clinically stable on a low dose of full-spectrum CBD-rich oil.	No evidence of DLE recurrence was observed.	Da Silva et al. 2024

N.R.: Not reported

In the only published randomized, placebo-controlled, blinded clinical trial aimed at determining the impact of capsules containing a CBD/CBDA-rich hemp oil (2–2.5 mg/kg orally twice daily for four weeks) on acute post-operative pain in dogs following tibial plateau levelling osteotomy (TPLO), pain scores (obtained by CBPI), degree of lameness, degree of weight-bearing, or radiographic healing of the osteotomy did not differed significantly between placebo and CBD/CBDA groups at any point, but an increase in alkaline phosphatase (ALP) activity was observed (Klatzkow et al. 2023). However, a previous study where a CBD/CBDA-rich hemp oil was given at 5 mg/kg in postsurgical intervertebral disc disease suggested lower postsurgical pain scores compared to placebo based on blinded veterinary assessment (Wright 2022).

Very recently, the anesthetic sparing effect of a single oral transmucosal full spectrum CBD-rich extract (6 mg/kg of total pCBs) has been demonstrated in 9 dogs. Indeed, a reduction of 23% on propofol dose necessary for induction was obtained in CBD treated dogs with respect to a placebo group, indicating that pCBs could be an adjunct option in anesthesia (Hasckel Gewehr et al. 2024).

The efficacy and tolerability of CBD in treating epilepsy in dogs have been investigated in three randomized, placebo-controlled, double-blinded clinical trials, and in one case series. The three randomized controlled clinical trials (Garcia et al. 2022; McGrath et al. 2019; Rozental et al. 2023b) produced promising results, as all studies showed a reduction in seizures frequency (total seizures or seizure days) in the CBD treated dogs compared to placebo. However, the case series by Mogi and Fukuyama (2019) reported different and sometimes contradictory results in the three evaluated dogs (Mogi and Fukuyama 2019) (see Table 2 for a detailed description of these studies).

It is noteworthy that in the study by Rozental et al. (2023b) the interactions between antiseizure drugs (ASD), such as phenobarbital, potassium bromide, zonisamide and levetiracetam, and CBD treatment did not affect the percentage change of total seizures or seizure days. Additionally, there was no evidence of differences between CBD administration and placebo regarding the percentage change from baseline in ASD concentrations for any of the measured ASDs (Rozental et al. 2023b). A previous study by Doran et al. (2022), which explored the drug-drug interactions between CBD and phenobarbital in healthy dogs, did not find any significant pharmacokinetic interactions between the two drugs. This indicates that, at least in healthy dogs, CBD and phenobarbital can be co-administered without notable alterations in their respective pharmacokinetics (Doran et al. 2022).

Five scientific studies on the clinical efficacy of CBD in treating behavioral disorders have been published so far: a replicated 4 × 4 Latin square design experiment (Morris et al. 2020), a placebo-controlled study (Corsetti et al. 2021), two blinded, placebo-controlled, parallel-design study (Hunt et al. 2023; Flint et al. 2024) and a pilot study (Masataka 2024). The efficacy of CBD treatment was evaluated in different stressful conditions, such as fear-inducing stimuli, shelter dogs, separation from caregivers ad car travels. In all studies but the one by Morris et al. (2020) (which results did not provide strong support for the anxiolytic effect of CBD in dogs), the efficacy of CBD in reducing stress in treated dogs was evidenced (see Table 2 for details).

Recently, four studies investigating the efficacy of CBD and CBD/CBDA as a treatment for canine skin diseases have also been published. They account for a randomised, double-blinded and placebo-controlled trial (Loewinger et al. 2022), a double-blinded and placebo-controlled trial (Mariga et al. 2023), a retrospective case series (Mogi et al. 2022) and a case report (Da Silva et al. 2024). The first three studies were aimed to evaluate the effect of CBD on lesion’s severity and pruritus in dogs affected by atopic dermatitis. While the severity of the lesions was not improved by CBD treatment in any of these three studies, an improvement in itching was observed in studies by Loewinger et al. (2022) and Mogi et al. (2022). It is noteworthy that in these studies the CBD-based solution was administered orally; meanwhile, humans’ studies have demonstrated a potential therapeutic value of CBD applied topically for skin diseases (Martins et al., 2022). The case report was instead intended to test the efficacy of a CBD treatment in a dog suffering with discoid lupus erythematosus (DLE) and resistant to conventional treatments. Cannabidiol was able to significantly improve skin lesions within a few weeks of treatment, and after 1 year the dog remained clinically stable on a low dose of CBD (Da Silva et al. 2024). Table 2 reports more detailed information on the studies cited above. 

A fifth study (a randomized complete block design, placebo-controlled study) intended to determine the influence of CBD (1.25 mg/kg or 2.5 mg/kg orally twice daily for three weeks) on the dogs’ daily activity, showed that CBD did not alter the total daily activity points or activity duration, but reduced total daily scratching compared with the control, albeit not statistically (Morris et al. 2021).

From this summary of studies, it is possible to conclude that some evidence exists supporting the beneficial role of CBD for adverse conditions in dogs, including OA pain, seizures, behavioral and skin problems. However, it is worth reporting the conclusions of two studies aimed to summarize the evidence of efficacy and safety of the use of cannabis for the treatment of animal disease and to assess the risk of bias regarding the obtained results. The first study (a systematic review of randomized clinical trials) included six trials meeting the inclusion criteria (i.e., Gamble et al. 2018; McGrath et al. 2019; Brioschi et al. 2020; Verrico et al. 2020; Corsetti et al. 2021; Mejia et al. 2021) and found that all of them presented a certain risk of bias (classified as low risk, some concern or high risk) accounting for one or more of the following items: randomization process, deviation from intended interventions, missing outcome data, measurement of the outcome, and selection of the reported results (Lima et al. 2022). The second study (a systematic review and meta-analysis of animal intervention studies) examined the results obtained by Gamble et al. 2018; Brioschi et al. 2020; Kogan et al. 2020; Verrico et al. 2020; and Mejia et al. 2021; addressing six types of bias: selection bias, performance bias, detection bias, attrition bias, reporting bias, and other, across 10 domains (with each domain categorized as high, unclear, or low risk of bias). Each of the identified study presented a certain level of bias, and the Authors concluded that the evidence is very uncertain to confirm the clinical efficacy of CBD treatment (Patikorn et al. 2023). Overall, both systematic reviews indicate that the results of published studies, despite being randomized, double-blinded, and placebo-controlled, should be interpreted with caution. Greater attention to study design and the definition and measurement of outcomes is needed in future research to strengthen the evidence regarding the therapeutic benefits of CBD for dogs.

In the matter of tolerability, in addition to the above studies (see Table 2 for tolerability results), other research has evaluated the safety of *Cannabis* derivatives, often together with their pharmacokinetics.

In the study by McGrath et al. (2018), diarrhea, vomiting, erythematous pinnae, transient isosthenuria, hyposthenuria or proteinuria, and increased ALP activity, nasal discharge, salivary staining, lameness, prolapsed nictitan and hyperthermia were observed after the administration of CBD oil, CBD microencapsulated and CBD cream (10 or 20 mg/kg) twice daily orally or transdermal for six weeks in 30 dogs. Loose stool and vomiting (food or bile products) were also reported by Deabold et al. (2019) after treatment with an oil (embedded in soft chews) containing 1 mg/kg of CBD and 1 mg/kg of CBDA, orally twice daily for 12 weeks in 8 dogs. Mild and self-limiting gastrointestinal signs (mainly hypersalivation) and transient increase of ALP were reported when a CBD hemp oil (1, 2, 4, or 12 mg/kg) was given orally once daily for four weeks in 20 dogs (Vaughn et al. 2021).

Increased ALP activity was also observed by Bradley et al. (2022), who administered CBD hemp oil at 4 mg/kg orally daily for 26 weeks in 40 subjects. Tittle et al. (2022) reported vomiting, loose stools and occasional episodes of licking, grimacing and chomping after oral treatment with 2 mg/kg CBD/CBDA-rich soft gel and hemp oil twice daily for four weeks in 8 dogs, while Doran et al. (2022) described the occurrence of vomiting, hyporexia, anorexia and an increase in serum ALP activity after the administration of 5, 10 or 20 mg/kg of pure CBD in MCT oil twice daily for two weeks in 9 dogs (3 x dosage).

In a study conducted by Chicoine and colleagues, neurological signs (head bobbing, hyperesthesia, ataxia or swaying, among others) were observed in six dogs treated with a 1:20 THC: CBD *Cannabis* herbal extract with a dosage of 10 mg/kg of CBD and 0,5 mg/kg of THC (Chicoine et al. 2020).

Additionally, a case report published by Simpson et al. (2020) described widespread cutaneous erythema and ulceration associated with anorexia and diarrhea in a dog. These symptoms occurred five days after the dog received an oral hemp oil formulation for anxiety, with a dosage of 0.3 mg/kg of CBD administered once daily (Simpson et al. 2020).

Very recently, the tolerability of oils containing different cannabinoids (broad spectrum CBD, broad spectrum CBD with CBG, or broad spectrum CBD with CBDA at 5 mg total cannabinoids/kg body weight/day) given orally was evaluated in a randomized, non-blinded, negative controlled, parallel design 90-day repeat dose study in 8 dogs/group. Clinical examinations, body weights, food consumption, serum hematology and biochemistry, coagulation parameters, and urinalysis were conducted at prefixed experimental times during the treatment period and up to 2 weeks after dosing. No somnolence, adverse effetcts (AEs) or serious AEs were reported during the study. The most common abnormal observation was diarrhea. Some hematology and clinical chemistry parameters showed statistically significant within-treatment group changes compared to baseline value. However, most of these changes were either transient or were within reference ranges. No significant abnormalities were recorded for any urinalysis parameters evaluated (Bookout et al. 2024).

The long-term tolerability of an industrial hemp broad spectrum extract with 94.5% CBD, 4.6% CBG, 0.3% CBDV, and 0.6% CBCH, administered orally to healthy dogs for 36 weeks at dosages of 5 and 10 mg/kg/day was also assessed in a randomized placebo-controlled study. Adverse events were recorded daily. Cell blood count and blood biochemistry profiles were monitored every 4 weeks, as well as physical examination (temperature, heart rate, respiratory rate, weight, and body fat index, observations of skin, eyes, nose, mouth and teeth, heart and lungs, the abdomen, lymph nodes, mentation and personality, activity level, and vocalization). The most prevalent AEs were gastrointestinal (soft feces or diarrhea with tenesmus and straining), and all observed AEs were mild. Dogs in the 10 mg/kg group had a higher frequency of soft feces than the 5 mg/kg group and placebo. All dogs dosed with both CBD oil doses had overall higher ALP activity than those given placebo, and monthly variations with a significant time effect were observed (Corsato Alvarenga et al. 2024).

Besides clinical trials, a preclinical/preregistration study was conducted in healthy male and females Beagle dogs to study the toxicology of a purified CBD extract (Epidiolex™). Dogs were treated over 39 weeks either with 0 mg/kg/day (control group C), 10, 50 and 100 mg/kg/day. Soft/liquid/mucoid feces, reduced body weight, marked increases in ALP (up to 8-fold compared to C), and liver changes, such as hepatocyte hypertrophy associated with increased liver weight and macroscopic enlargement, were observed at all doses, as well as consistent decreases in heart rate in males at the higher dose (Food and Drug Administration Application 2018).

From the above findings, an oral administration of CBD 2 mg/kg once daily and up to 20 mg/kg/ twice daily appeared well-tolerated with mild side effects, observed in both healthy and diseased animals. This favorable safety profile aligns with the findings of a study conducted by Vaughn et al. (2020), which utilized increasing oral doses of CBD, reaching up to 62 mg/kg (Vaughn et al. 2020).

To the best of the authors’ knowledge, only one paper has been published so far on the physiological effect of CBG and its acid derivative (CBGA) in dogs. Following an oral twice-daily administration of an equal mix of CBG and CBGA at a dosage of 2 mg/kg for two weeks in 6 fasted and fed dogs, physical examination, comprehensive blood analyses, and serum chemistry assessments conducted throughout the two weeks indicated the absence of any adverse events during this short-term dosing trial. Indeed, notwithstanding some changes between baseline and week 2, in some cases statistically significant, no values for any parameters were outside of the normal reference range established by the Cornell Veterinary Diagnostic Laboratory Clinical Pathology Services, and physical examinations performed throughout the treatment period found no observable abnormalities regarding activity, neurological deficits, or behavior at any stage of the study. Interestingly, rises in liver enzymes were not observed in this study. ALP, in particular, decreased in both the fasted and fed states (Amstutz et al. 2022).

### Cats

Compared to dogs, there is a significantly smaller number of scientific studies concerning the use of *Cannabis* derivatives in cats. However, some information can be found even for this animal species, with *Cannabis* derivatives tested in a placebo-controlled trial evaluating the effectiveness and safety of CBD for pain management in feline chronic gingivostomatitis (FCGS) (Coelho et al. 2023), and in a case report on a cat with chronic osteoarthritic pain (Gutierre et al. 2023).

In the first study, CBD was administered orally within a multimodal treatment (including butorphanol and meloxicam) in cats with FCGS undergoing partial or total dental extractions. The intervention was positively associated with improved Stomatitis Disease Activity Index (SDAI) score and a marked relief of oral pain after the surgery using the Composite Oral Pain Scale Canine/Feline (COPS-C/F) (Coelho et al. 2023).

In the case report on a cat with osteoarthritis, pain scored with the Feline Musculoskeletal Pain Index (FMPI) decreased of 8 points, from 13 on day 0 (before treatment) to 5 points on day 30 (Gutierre et al. 2023).

More details on the two cited studies are reported in Table 3).

**Table 3:** Summary of studies on the efficacy of *Cannabis* derivatives in cats

Study design, formulation, dose, treatment duration and *n*. of recruited cats	Efficacy	Tolerability	Reference
Cannabis derivatives in chronic pain from chronic gingivostomatitis			
Placebo-controlled trial to evaluate the effectiveness of a commercially available CBD oral formulation as an add-on treatment for pain management in 22 cats with chronic gingivostomatitis (FCGS). CBD was administered within a multimodal treatment (including butorphanol and meloxicam) in cats with FCGS undergoing partial or total dental extractions. Cats received a fixed dosage of 4 mg/cat CBD or placebo twice daily from two hours before the dental extractions and for the following 15 days.	Cats treated with CBD significantly improved the Stomatitis Disease Activity Index (SDAI) score, with 2.6 points less than the placebo group at the end of the 15-day treatment. Both groups showed a marked relief of oral pain after surgery using the Oral Pain Scale Canine/Feline (COPS-C/F): the CBD group scored an average of 3 points less on the scale compared with the placebo group, although this difference was not statistically significant.	Clinician reported salivation, licking and head shaking after CBD administration in 5 cats, while owners reported diarrhea (1 cat) and vomiting (2 cats). Only a not significant increase of aspartate transaminase (AST) and albumin was recorded in the CBD group compared to placebo.	Coelho et al. 2023
*Cannabis* derivatives in chronic pain from osteoarthritis (OA)			
Case report on a cat with chronic OA pain treated with a full spectrum cannabis oil (1.8% CBD and 0.8% THC) at 0.5 mg/kg (based on CBD) orally twice daily for the first two days. Because of the occurrence of sedation, the dose was decreased to 0.25 mg/kg twice daily for the following four weeks.	Decrease of pain scores measured by the Feline Musculoskeletal Pain Index (FMPI) from 13 on day 0 (before treatment) to 5 points on day 30.	The alanine aminotransferase (ALT) increased of approximately 3.2 times with 30-day treatment.	Gutierre et al. 2023

When tolerability was assessed within a pharmacokinetic study conducted in 8 healthy cats dosed orally with a CBD-infused fish oil (50/50% mix of CBD and CBDA) at 2 mg/kg twice daily for 84 days, no changes in physical examination and few changes in the mean cell counts (slight decrease in eosinophil counts) and serum chemistry parameters (a single cat with elevated alanine aminotransferase (ALT) level during treatment, a decrease in Blood Urea Nitrogen, triglycerides and creatine kinase activity over time) were observed, suggesting the relative safety of the oral supplementation over 12 weeks. Cats commonly displayed excessive licking and head shaking with oil administration (Deabold et al. 2019).

Two years later, Kulpa et al. (2021) evaluated the safety and tolerability of up to 11 escalating doses of orally delivered cannabis oils predominant in CBD, THC, or both CBD and THC in 20 healthy cats in a placebo-controlled, blinded study. Clinical observations, complete blood counts (CBCs) and clinical chemistry were evaluated as outcomes. All cats safely reached the highest doses of 30.5 mg/kg CBD, 41.5 mg/kg THC, or 13.0:8.4 mg/kg CBD: THC. Any observed AEs were mild, temporary, and resolved without medical intervention. Constitutional symptoms like lethargy and hypothermia, as well as neurological symptoms such as ataxia and ocular symptoms like protrusion membrana nictitans, were more prevalent with oils containing THC (both CBD/THC and THC oils). There were no significant changes in hematology or clinical chemistry observed among the different treatment groups (Kulpa et al. 2021).

Recently, Coltherd et al. (2024) published the results of a randomized, blinded, and placebo-controlled study with CBD or placebo administered orally at 4 mg/kg twice daily for 26 weeks in 10 cats/group. All biochemistry and hematology data showed no clinically significant differences between supplement group but ALT, for which a statistical equivalence (at 2-fold limits) was found.

### Horses

To the best of the authors’ knowledge, only three clinical studies on the efficacy of CBD in horses are available so far, with information detailed in Table 4.

**Table 4:** Summary of studies on the efficacy of *Cannabis* derivatives in horses

Study design, formulation, dose, treatment duration and *n*. of recruited horses	Efficacy	Tolerability	Reference
Cannabis derivatives in sensitivity to touch			
Case study on a 4-year-old Quarter Horse mare with a 5-week history of strong sensitivity to touch near the withers/shoulder region not responding to conventional therapeutic approaches (dexamethasone, gabapentin, magnesium/vitamin E, prednisolone and aquapuncture with vitamin B12), to evaluate the efficacy of a pure crystalline CBD formulation on allodynia. Initial CBD dose was 250 mg orally twice daily for 60 days. The dose was then decreased by one-half, then again adjusted to the initial level, and gradually decreased during two following months, reaching a maintenance dose of 150 mg once a day.	After the 250 mg dose the condition significantly improved 36 h after treatment beginning.	**N.R.**	Ellis and Contino 2021
	The reduction of the CBD dose resulted in a recurrence of the clinical signs after one day. With the reinstatement of the initial dose and its following gradual reduction, no repeated presence of increased sensitivity was evidenced.		
	The owner described a 90% improvement.		
*Cannabis* derivatives in crib-biting and wind-sucking			
Case report on a 22-year-old mare suffering from chronic crib-biting and wind-sucking started at the age of 7 years old and progressively worsened over the years to evaluate the efficacy of isolated CBD (0.5 mg/kg orally twice a day for four weeks).	Between the first and second weeks of treatment a significant decrease in the hours spent crib-biting and wind-sucking was observed, with a gradual and constant enhancement of appetite.	No adverse events were reported among those carefully monitored (colic, lethargy, inappetence, hyperthermia, diarrhea, sialorrhea, cardiorespiratory disturbances, and ataxia).	Cunha et al. 2023
*Cannabis* derivatives in chronic pain from osteoarthritis (OA)			
Randomized, placebo-controlled study to evaluate the efficacy of a commercial hemp oil containing 15% CBD, administered OTM at a dose of 0.03mg/kg every 24 h for 2 weeks, in relieving pain in 12 horses with osteoarthritis The CBD treatment was included in a classical pharmacological regimen, witha control group treated with the traditional analgesic (phenylbutazone) only.	A significant reduction in the Horse Chronic Pain Scale (HCPS) scores was seen in both groups. However, lower HCPS scores were recorded in the CBD group. A significant reduction in heart rate, respiratory rate, white blood cell counts and oxidative stress was also recorded.	The addition of a CBD-based product to the analgesic protocol was well tolerated, as no adverse effects were observed.	Interlandi et al. 2024

N.R.: Not reported

Ellis and Contino (2021) published a case study concerning a 4-year-old Quarter Horse mare with a 5-week history of strong sensitivity to touch near the withers/shoulder region, probably due to an insect bite. Conventional therapeutic approaches did not improve the clinical signs. After treatment with CBD, the condition significantly improved after 36 h. An attempt to reduce the dose by half was made, resulting in a recurrence of the clinical signs after one day. The dose was, therefore, adjusted to the initial level, and gradually decreased during two months without repeated presence of increased sensitivity and the owner describing a 90% improvement (Ellis and Contino 2021).

Another case report was published by (Cunha et al. 2023), who described a 22-year-old mare suffering from chronic crib-biting and wind-sucking that started at the age of 7 years old and got progressively worse over the years, and the successful outcome of four weeks-therapy with CBD (Cunha et al. 2023).

Recently, the efficacy of CBD oil in relieving pain in 12 horses with osteoarthritis was also evaluated in a randomized study (Interlandi et al. 2024). The CBD treatment was included in a classical pharmacological regimen, with a control group treated with the traditional analgesic (phenylbutazone) only. A significant reduction in the Horse Chronic Pain Scale (HCPS) scores was seen in both groups. However, lower HCPS scores were recorded in the CBD group (Interlandi et al. 2024).

Moreover, in 2020, a patent application (US 10,624,936 B2) concerning the use of CBD in stress and anxiety disorders in horses described the calming effects of CBD in 7 horses of different ages and sexes, where a single administration of a water-based CBD formulation at doses of 50 or 100 mg/horse (depending on the size of the animal) dramatically improved the behavior associated with stress or anxiety (Denapoli and Denapoli 2020).

Besides the described clinical studies, a preliminary experimental study was also conducted (McIver et al. 2020). The study was aimed to evaluate the effect of a 1% CBD extract in 2 mL of manuka honey applied topically daily for 42 days, compared to the sole manuka honey, on second intention wound healing in distal limb wounds of 6 horses. The study failed to demonstrate any difference in wound healing variables (wound area, daily healing rate, days to complete healing) between the two treatment groups (McIver et al. 2020).

Finally, it’s worth mentioning the study by Turner et al. (2023), who evaluated the effects of a broad spectrum hemp extract (98% CBD) on immune function (by measuring inflammatory cytokines and antibody responses to vaccination) and health parameters in senior horses. Horses were vaccinated with an equine influenza vaccine and orally-dosed with CBD (2 mg/kg: 13 horses) or control (soy oil: 14 horses) daily for 90 days. A significant decrease was determined for whole blood inflammatory cytokine expression of IFN-γ at day 60, and for IL6 at day 60 and 90 in CBD-treated horses when compared to control horses. CBD did not significantly affect any other immune factors, hemagglutination inhibition titers, or health parameters (Turner et al. 2023).

In the matter of safety, CBD has been referred to as a generally well-tolerated substance in horses (Draeger et al. 2021a; St. Blanc et al. 2022). Indeed, the blinded, placebo-controlled study conducted by St. Blanc et al. (2022), where daily oral supplementation of CBD (150 mg) was given for 56 days in 10 horses, did not reveal any difference from placebo regarding blood count, biochemical panel, as well as sedation and ataxia. Similarly, in horses (n. 30) randomly treated orally with CBD (0.75 or 150 mg/kg) or placebo twice daily for 28 days, body weight, body condition score, and blood chemical parameters were not adversely affected following supplementation (Leise et al. 2023).

Mild hypocalcemia was seen in all horses (n. 12), and elevated liver enzymes were observed in 8/12 horses after administration of CBD (0.5–1.5 mg/kg orally twice daily for six weeks). However, these changes improved or normalized within ten days after the final CBD dose (Yocom et al. 2022).

More recently, Eichler and co-workers performed two randomized, blinded, placebo-controlled studies where CBD was provided as a single oral administration at escalating doses (0.2, 1 and 3 mg/kg in 3, 3 and 5 horses, respectively) (study 1), and as multiple oral administration (3 mg/kg twice daily for 15 days in 6 horses) (study 2). In the first study, behavioral parameters, as measured using the FaceSed (facial sedation scale for horses) and the Horse Grimace Scale, as well as heart rate (HR) and heart rate variability (HRV), were evaluated at 0, 1, 2, 4 and 12 h after CBD or placebo administration. In the second study, blood and saliva cortisol were also measured throughout the study period besides behavioral and heart parameters. Both the single and multiple CBD administration trials revealed no statistically significant effect in horses’ behavioral observations, HR and HRV, and cortisol levels (Eichler et al. 2023a, 2024). Draeger and co-workers previously obtained similar findings on HR after administering 100 mg of CBD or placebo once daily for six weeks. However, treated horses exhibited less reactivity after six weeks of supplementation, suggesting that CBD supplementation may lower reactivity in horses (Draeger et al. 2021b).

### Cattle

Only one study is available so far where a cannabis derivative has been evaluated in the bovine species. In this study, 8 cattle were fed with industrial hemp (IH) once a day for 14 days, receiving a target daily dose of 5.5 mg/kg of CBDA, and behavior, serum cortisol, serum haptoglobin, liver enzymes, serum amyloid A, and prostaglandin E2 concentrations were evaluated compared with a control (CNTL) group. The IH group showed increased lying behavior compared to the CNTL group. Cortisol, and prostaglandin E2 concentrations were lower in the IH than the CNTL group, and no differences for haptoglobin or serum amyloid A were observed. Authors thus suggested that feeding IH with a high CBDA content for 14 days increases lying behavior and decreases biomarkers of stress and inflammation in cattle (Kleinhenz et al. 2022).

## Conclusion

The discovery of the ECS and the recognition of its involvement in multiple physiological processes opened the scenario of a possible modulation of its functions using exogenous cannabinoids as therapeutic intervention.

For instance, the involvement of the dorsal root ganglia in chronic pain and the expression of CB receptors at this level, allow to hypothesize a role of modulation of their activity in the management of animal pain (Chiocchetti et al. 2019, 2021; Galiazzo et al. 2022). The different expression disease-correlated of CB1 receptors in dogs with idiopathic and structural epilepsy may be noteworthy in the development of specific therapeutic protocols for dogs (Kostic et al. 2023). Similarly, an intervention on ECS could be hypothesized to manage feline chronic gingivostomatitis or dermatitis of dogs and cats with hypersensitivity, by virtue of the overexpression of cannabinoids receptors in these subjects compared to healthy ones (Campora et al. 2012; Miragliotta et al. 2018; Polidoro et al. 2021; Chiocchetti et al. 2022a, b).

However, even if there are some evidences of a beneficial role of pCBs for adverse conditions in animals, the results obtained so far are still not always consistent because of some limitations (e.g. small sample size, concomitant administration of other drugs, subjective evaluations by owners and veterinarians as well as the large variability of the pCBs content in the used formulations, etc.) (Di Salvo et al. 2023).

It is therefore clear that further investigations need to better understand and identify how possible interventions at the ECS level can be exploited in veterinary therapy.

## Data Availability

No datasets were generated or analysed during the current study.

## References

[CR1] Alonso-Ferrero ME, Paniagua MA, Mostany R, Pilar-Cuéllar F, Díez-Alarcia R, Pazos A, Fernández-López A (2006) Cannabinoid system in the budgerigar brain. Brain Res 1087:105–113. 10.1016/j.brainres.2006.02.119 16626655 10.1016/j.brainres.2006.02.119

[CR2] Amstutz K, Schwark WS, Zakharov A, Gomez B, Lyubimov A, Ellis K, Venator KP, Wakshlag JJ (2022) Single dose and chronic oral administration of cannabigerol and cannabigerolic acid-rich hemp extract in fed and fasted dogs: physiological effect and pharmacokinetic evaluation. J Vet Pharmacol Ther 45:245–254. 10.1111/jvp.13048 35246858 10.1111/jvp.13048

[CR3] Anderson LL, Low IK, Banister SD, McGregor IS, Arnold JC (2019) Pharmacokinetics of phytocannabinoid acids and anticonvulsant effect of cannabidiolic acid in a mouse model of Dravet syndrome. J Nat Prod 82:3047–3055. 10.1021/acs.jnatprod.9b00600 31686510 10.1021/acs.jnatprod.9b00600

[CR4] Anderson LL, Etchart MG, Bahceci D, Golembiewski TA, Arnold JC (2021) Cannabis constituents interact at the drug efflux pump BCRP to markedly increase plasma cannabidiolic acid concentrations. Sci Rep 11:14948. 10.1038/s41598-021-94212-6 34294753 10.1038/s41598-021-94212-6 PMC8298633

[CR5] Ang SP, Sidharthan S, Lai W, Hussain N, Patel KV, Gulati A, Henry O, Kaye AD, Orhurhu V (2023) Cannabinoids as a potential alternative to opioids in the management of various pain subtypes: benefits, limitations, and risks. Pain Ther 12:355–375. 10.1007/S40122-022-00465-Y 36639601 10.1007/s40122-022-00465-y PMC10036719

[CR6] Atalay Ekiner S, Gęgotek A, Skrzydlewska E (2022) The molecular activity of cannabidiol in the regulation of Nrf2 system interacting with NF-κB pathway under oxidative stress. Redox Biol 57:102489. 10.1016/j.redox.2022.102489 36198205 10.1016/j.redox.2022.102489 PMC9535304

[CR7] Bartner LR, McGrath S, Rao S, Hyatt LK, Wittenburg LA (2018) Pharmacokinetics of cannabidiol administered by 3 delivery methods at 2 different dosages to healthy dogs. Can J Vet Res 82:178–183 30026641 PMC6038832

[CR8] Berthold EC, Kamble SH, Kanumuri SRR, Kuntz MA, Senetra AS, Chiang Y-H, McMahon LR, McCurdy CR, Sharma A (2023) Comparative pharmacokinetics of commercially available cannabidiol isolate, broad-spectrum, and full-spectrum products. Eur J Drug Metab Pharmacokinet 48:427–435. 10.1007/s13318-023-00839-3 37337087 10.1007/s13318-023-00839-3

[CR9] Bie B, Wu J, Foss JF, Naguib M (2018) An overview of the cannabinoid type 2 receptor system and its therapeutic potential. Curr Opin Anaesthesiol 31:407–414. 10.1097/ACO.0000000000000616 29794855 10.1097/ACO.0000000000000616 PMC6035094

[CR10] Blebea NM, Pricopie AI, Vlad RA, Hancu G (2024) Phytocannabinoids: exploring pharmacological profiles and their impact on therapeutical use. Int J Mol Sci 25. 10.3390/IJMS25084204 10.3390/ijms25084204 PMC11050509 38673788

[CR11] Bookout W, Dziwenka M, Valm K, Kovacs-Nolan J (2024) Safety study of cannabidiol products in healthy dogs. Front Vet Sci 11:1349590. 10.3389/fvets.2024.1349590 38496308 10.3389/fvets.2024.1349590 PMC10940325

[CR12] Bradley S, Young S, Bakke AM, Holcombe L, Waller D, Hunt A, Pinfold K, Watson P, Logan DW (2022) Long-term daily feeding of cannabidiol is well-tolerated by healthy dogs. Front Vet Sci 9:977457. 10.3389/fvets.2022.977457 10.3389/fvets.2022.977457 PMC9533147 36213402

[CR13] Brioschi FA, Di Cesare F, Gioeni D, Rabbogliatti V, Ferrari F, D’Urso ES, Amari M, Ravasio G (2020) Oral transmucosal cannabidiol oil formulation as part of a multimodal analgesic regimen: effects on pain relief and quality of life improvement in dogs affected by spontaneous osteoarthritis. Animals 10:1505. 10.3390/ani10091505 32858828 10.3390/ani10091505 PMC7552307

[CR14] Brown C, Mitsch M, Blankenship K, Campbell C, Pelanne M, Sears J, Bell A, Olivier AK, Ross MK, Archer T, Kaplan BLF (2023) Canine immune cells express high levels of CB1 and CB2 cannabinoid receptors and cannabinoid-mediated alteration of canine cytokine production is vehicle-dependent. Vet Immunol Immunopathol 265:110667. 10.1016/j.vetimm.2023.110667 37931433 10.1016/j.vetimm.2023.110667 PMC11798033

[CR15] Campora L, Miragliotta V, Ricci E, Cristino L, Di Marzo V, Albanese F, Federica Della Valle M, Abramo F (2012) Cannabinoid receptor type 1 and 2 expression in the skin of healthy dogs and dogs with atopic dermatitis. Am J Vet Res 73:988–995. 10.2460/ajvr.73.7.988 22738050 10.2460/ajvr.73.7.988

[CR16] Carlisle SJ, Marciano-Cabral F, Staab A, Ludwick C, Cabral GA (2002) Differential expression of the CB2 cannabinoid receptor by rodent macrophages and macrophage-like cells in relation to cell activation. Int Immunopharmacol 2:69–82. 10.1016/S1567-5769(01)00147-3 11789671 10.1016/s1567-5769(01)00147-3

[CR17] Chicoine A, Illing K, Vuong S, Pinto KR, Alcorn J, Cosford K (2020) Pharmacokinetic and safety evaluation of various oral doses of a novel 1:20 THC:CBD cannabis herbal extract in dogs. Front Vet Sci 7. 10.3389/fvets.2020.583404 10.3389/fvets.2020.583404 PMC7550466 33134364

[CR18] Chiocchetti R, Galiazzo G, Tagliavia C, Stanzani A, Giancola F, Menchetti M, Militerno G, Bernardini C, Forni M, Mandrioli L (2019) Cellular distribution of canonical and putative cannabinoid receptors in canine cervical dorsal root ganglia. Front Vet Sci 6:313. 10.3389/fvets.2019.00313 10.3389/fvets.2019.00313 PMC6761858 31608295

[CR19] Chiocchetti R, Rinnovati R, Tagliavia C, Stanzani A, Galiazzo G, Giancola F, Silva M, De, Capodanno Y, Spadari A (2021) Localisation of cannabinoid and cannabinoid-related receptors in the equine dorsal root ganglia. Equine Vet J 53:549–557. 10.1111/evj.13305 32524649 10.1111/evj.13305

[CR20] Chiocchetti R, De Silva M, Aspidi F, Cunha RZ, Gobbo F, Tagliavia C, Sarli G, Morini M (2022a) Distribution of cannabinoid receptors in keratinocytes of healthy dogs and dogs with atopic dermatitis. Front Vet Sci 9:915896. 10.3389/fvets.2022.915896 10.3389/fvets.2022.915896 PMC9305491 35873682

[CR21] Chiocchetti R, Salamanca G, De Silva M, Gobbo F, Aspidi F, Cunha RZ, Galiazzo G, Tagliavia C, Sarli G, Morini M (2022b) Cannabinoid receptors in the inflammatory cells of canine atopic dermatitis. Front Vet Sci 9:987132. 10.3389/fvets.2022.987132 36187821 10.3389/fvets.2022.987132 PMC9521433

[CR22] Coelho RCMP, De OP, Leme F, Moreira A, Branco FEMT, Melo SM, De Melo MG, E (2021) Current review of hemp-based medicines in dogs. J Vet Pharmacol Ther 44:870–882. 10.1111/jvp.13016 34605042 10.1111/jvp.13016

[CR23] Coelho JC, Duarte N, Da Silva B, Bronze A, Mestrinho MDR, L.A (2023) Placebo-controlled trial of daily oral cannabidiol as adjunctive treatment for cats with chronic gingivostomatitis. Animals 13:2716. 10.3390/ani13172716 37684980 10.3390/ani13172716 PMC10487179

[CR24] Coltherd JC, Bednall R, Bakke AM, Ellerby Z, Newman C, Watson P, Logan DW, Holcombe LJ (2024) Healthy cats tolerate long-term daily feeding of Cannabidiol. Front Vet Sci 10:1324622. 10.3389/fvets.2023.1324622 38327816 10.3389/fvets.2023.1324622 PMC10847353

[CR25] Console-Bram L, Brailoiu E, Brailoiu GC, Sharir H, Abood ME (2014) Activation of GPR18 by cannabinoid compounds: a tale of biased agonism. Br J Pharmacol 171:3908–3917. 10.1111/bph.12746 24762058 10.1111/bph.12746 PMC4128052

[CR26] Corsato Alvarenga I, Gustafson D, Banks K, Wilson K, McGrath S (2023) Cannabidiol plasma determination and pharmacokinetics conducted at beginning, middle and end of long-term supplementation of a broad-spectrum hemp oil to healthy adult dogs. Front Vet Sci 10:1279926. 10.3389/fvets.2023.1279926 37841465 10.3389/fvets.2023.1279926 PMC10571049

[CR27] Corsato Alvarenga I, Wilson KM, McGrath S (2024) Tolerability of long-term cannabidiol supplementation to healthy adult dogs. J Vet Intern Med 38:326–335. 10.1111/jvim.16949 38009749 10.1111/jvim.16949 PMC10800185

[CR28] Corsetti S, Borruso S, Malandrucco L, Spallucci V, Maragliano L, Perino R, DAgostino P, Natoli E (2021) Cannabis sativa L. may reduce aggressive behaviour towards humans in shelter dogs. Sci Rep 11:2773. 10.1038/s41598-021-82439-2 33531559 10.1038/s41598-021-82439-2 PMC7854708

[CR29] Costa B, Giagnoni G, Franke C, Trovato AE, Colleoni M (2004) Vanilloid TRPV1 receptor mediates the antihyperalgesic effect of the nonpsychoactive cannabinoid, cannabidiol, in a rat model of acute inflammation. Br J Pharmacol 143:247–250. 10.1038/sj.bjp.0705920 15313881 10.1038/sj.bjp.0705920 PMC1575333

[CR30] Court MH, Mealey KL, Burke NS, Jimenez TP, Zhu Z, Wakshlag JJ (2024) Cannabidiol and cannabidiolic acid: preliminary in vitro evaluation of metabolism and drugdrug interactions involving canine cytochrome P-450, UDP-glucuronosyltransferase, and P-glycoprotein. J Vet Pharmacol Ther 47:1–13. 10.1111/jvp.13403 37469115 10.1111/jvp.13403

[CR31] Cui Sun M, Otálora-Alcaraz A, Prenderville JA, Downer EJ (2024) Toll-like receptor signalling as a cannabinoid target. Biochem Pharmacol 222:116082. 10.1016/j.bcp.2024.116082 38438052 10.1016/j.bcp.2024.116082

[CR32] Cunha RZ, Felisardo LL, Salamanca G, Marchioni GG, Neto OI, Chiocchetti R (2023) The use of cannabidiol as a novel treatment for oral stereotypic behaviour (crib-biting) in a horse. Vet Anim Sci 19:100289. 10.1016/j.vas.2023.100289 36824298 10.1016/j.vas.2023.100289 PMC9941357

[CR33] Da Silva MES, Christianetti B, Amazonas E, Pereira ML (2024) Case report: cannabinoid therapy for discoid lupus erythematosus in a dog. Front Vet Sci 11:1309167. 10.3389/fvets.2024.1309167 38406630 10.3389/fvets.2024.1309167 PMC10884172

[CR34] Dall’Aglio C, Mercati F, Pascucci L, Boiti C, Pedini V, Ceccarelli P (2010) Immunohistochemical localization of CB1 receptor in canine salivary glands. Vet Res Commun 34:9–12. 10.1007/s11259-010-9379-0 10.1007/s11259-010-9379-0 20437096

[CR35] Dall’Aglio C, Polisca A, Cappai MG, Mercati F, Troisi A, Pirino C, Scocco P, Maranesi M (2017) Immunohistochemistry detected and localized cannabinoid receptor type 2 in bovine fetal pancreas at late gestation. Eur J Histochem. 10.4081/ejh.2017.2761 28348424 10.4081/ejh.2017.2761 PMC5364978

[CR36] Dawidowicz AL, Olszowy-Tomczyk M, Typek R, ∆9-THC CBGCBD (2021) CBN, CBGA, CBDA and ∆9-THCA as antioxidant agents and their intervention abilities in antioxidant action. Fitoterapia 152:104915. 10.1016/j.fitote.2021.104915 10.1016/j.fitote.2021.104915 33964342

[CR37] Deabold KA, Schwark WS, Wolf L, Wakshlag JJ (2019) Single-dose pharmacokinetics and preliminary safety assessment with use of CBD-Rich hemp nutraceutical in healthy dogs and cats. Animals 9:832. 10.3390/ani9100832 31635105 10.3390/ani9100832 PMC6826847

[CR38] Deiana S, Watanabe A, Yamasaki Y, Amada N, Arthur M, Fleming S, Woodcock H, Dorward P, Pigliacampo B, Close S, Platt B, Riedel G (2012) Plasma and brain pharmacokinetic profile of cannabidiol (CBD), cannabidivarine (CBDV), ∆9-tetrahydrocannabivarin (THCV) and cannabigerol (CBG) in rats and mice following oral and intraperitoneal administration and CBD action on obsessivecompulsive behaviour. Psychopharmacology 219:859–873. 10.1007/s00213-011-2415-0 21796370 10.1007/s00213-011-2415-0

[CR39] Della Rocca G, Di Salvo A (2020) Hemp in veterinary medicine: from feed to drug. Front Vet Sci 7:387. 10.3389/fvets.2020.00387 32850997 10.3389/fvets.2020.00387 PMC7399642

[CR40] Della Rocca G, Paoletti F, Conti MB, Galarini R, Chiaradia E, Sforna M, Dall’Aglio C, Polisca A, Di Salvo A (2023) Pharmacokinetics of cannabidiol following single oral and oral transmucosal administration in dogs. Front Vet Sci 9:1104152. 10.3389/fvets.2022.1104152 36686155 10.3389/fvets.2022.1104152 PMC9859632

[CR41] Denapoli A, Denapoli C (2020) Method of reducing stress and anxiety in equines. United States Patent. 2020 Apr; Patent No.: US 10,624,936 B2. Available at: https://patentimages.storage.googleapis.com/0c/8d/25/85f7c9a228d459/US10624936.pdf. Accessed 15 July 2024

[CR42] Deng J, Zhu X, Chen Z, Fan CH, Kwan HS, Wong CH, Shek KY, Zuo Z, Lam TN (2017) A review of fooddrug interactions on oral drug absorption. Drugs 77:1833–1855. 10.1007/s40265-017-0832-z 29076109 10.1007/s40265-017-0832-z

[CR43] Di Salvo A, Conti MB, della Rocca G (2023) Pharmacokinetics, efficacy, and safety of cannabidiol in dogs: an update of current knowledge. Front Vet Sci 10. 10.3389/fvets.2023.1204526 10.3389/fvets.2023.1204526 PMC10347378 37456953

[CR44] Di Wang MV J (2015) The endocannabinoidome: the world of endocannabinoids and related mediators. Academic Press

[CR45] Divín D, Goméz Samblas M, Kuttiyarthu Veetil N, Voukali E, Świderská Z, Krajzingrová T, Těšický M, Beneš V, Elleder D, Bartoš O, Vinkler M (2022) Cannabinoid receptor 2 evolutionary gene loss makes parrots more susceptible to neuroinflammation. Proc R Soc B Biol Sci 289:20221941. 10.1098/rspb.2022.1941 10.1098/rspb.2022.1941 PMC9727682 36475439

[CR46] Doran CE, McGrath S, Bartner LR, Thomas B, Cribb AE, Gustafson DL (2022) Drug-drug interaction between cannabidiol and phenobarbital in healthy dogs. Am J Vet Res 83:86–94. 10.2460/ajvr.21.08.0120 10.2460/ajvr.21.08.0120 34727050

[CR47] Draeger AL, Thomas EP, Jones KA, Davis AJ, Porr CAS (2021b) The effects of pelleted cannabidiol supplementation on heart rate and reaction scores in horses. J Veterinary Behav 46:97–100. 10.1016/j.jveb.2021.09.003

[CR48] Draeger AL, Hoffman LK, Godwin PR, Davis AJ, Porr SA, Draeger AL, Hoffman LK, Godwin PR, Davis AJ (2021a) Pharmacokinetics of a single feeding of pelleted cannabidiol in horses. Steeplechase: an ORCA Student Journal 4

[CR49] Eichler F, Ehrle A, Jensen KC, Baudisch N, Petersen H, Bäumer W, Lischer C, Wiegard M (2023a) Behavioral observations, heart rate and heart rate variability in horses following oral administration of a cannabidiol containing paste in three escalating doses (part 1/2). Front Vet Sci 10:1305868. 10.3389/fvets.2023.1305868 10.3389/fvets.2023.1305868 PMC10750369 38149295

[CR50] Eichler F, Poźniak B, Machnik M, Schenk I, Wingender A, Baudisch N, Thevis M, Bäumer W, Lischer C, Ehrle A (2023b) Pharmacokinetic modelling of orally administered cannabidiol and implications for medication control in horses. Front Vet Sci 10:1234551. 10.3389/fvets.2023.1234551 10.3389/fvets.2023.1234551 PMC10445762 37621871

[CR51] Eichler F, Ehrle A, Machnik M, Jensen KC, Wagner S, Baudisch N, Bolk J, Pötzsch M, Thevis M, Bäumer W, Lischer C, Wiegard M (2024) Behavioral observations, heart rate and cortisol monitoring in horses following multiple oral administrations of a cannabidiol containing paste (part 2/2). Front Vet Sci 10:1305873. 10.3389/fvets.2023.1305873 10.3389/fvets.2023.1305873 PMC10791836 38234983

[CR52] Ellis KL, Contino EK (2021) Treatment using cannabidiol in a horse with mechanical allodynia. Equine Vet Educ 33. 10.1111/eve.13168

[CR53] Etemad L, Karimi G, Alavi MS, Roohbakhsh A (2022) Pharmacological effects of cannabidiol by transient receptor potential channels. Life Sci 300. 10.1016/J.LFS.2022.120582 10.1016/j.lfs.2022.120582 35483477

[CR54] Fernández-Trapero M, Pérez-Díaz C, Espejo-Porras F, De Lago E, Fernández-Ruiz J (2020) Pharmacokinetics of Sativex^®^ in dogs: towards a potential cannabinoid-based therapy for canine disorders. Biomolecules 10:279. 10.3390/biom10020279 32054131 10.3390/biom10020279 PMC7072526

[CR55] Flint HE, Hunt ABG, Logan DW, King T (2024) Daily dosing of cannabidiol (CBD) demonstrates a positive effect on measures of stress in dogs during repeated exposure to car travel. J Anim Sci 102:skad414. 10.1093/jas/skad414 10.1093/jas/skad414 PMC10810271 38244994

[CR56] Food and Drug Administration application 210365Orig1s000 (2018) GW Pharmaceuticals. Available at: https://www.accessdata.fda.gov/drugsatfda_docs/nda/2018/210365Orig1s000PharmR.pdf. https://www.accessdata.fda.gov/drugsatfda_docs/nda/2018/210365Orig1s000SumR.pdf. Accessed 15 July 2024

[CR57] Fraguas-Sánchez AI, Martín‐Sabroso C, Torres‐Suárez AI (2018) Insights into the effects of the endocannabinoid system in cancer: a review. Br J Pharmacol 175:2566–2580. 10.1111/bph.14331 10.1111/bph.14331 PMC6003657 29663308

[CR58] Freundt-Revilla J, Kegler K, Baumgärtner W, Tipold A (2017) Spatial distribution of cannabinoid receptor type 1 (CB1) in normal canine central and peripheral nervous system. PLoS ONE 12:e0181064. 10.1371/journal.pone.0181064 10.1371/journal.pone.0181064 PMC5507289 28700706

[CR59] Galiazzo G, Giancola F, Stanzani A, Fracassi F, Bernardini C, Forni M, Pietra M, Chiocchetti R (2018) Localization of cannabinoid receptors CB1, CB2, GPR55, and PPARα in the canine gastrointestinal tract. Histochem Cell Biol 150:187–205. 10.1007/s00418-018-1684-7 10.1007/s00418-018-1684-7 29882158

[CR60] Galiazzo G, Tagliavia C, Giancola F, Rinnovati R, Sadeghinezhad J, Bombardi C, Grandis A, Pietra M, Chiocchetti R (2021) Localisation of cannabinoid and cannabinoid-related receptors in the horse ileum. J Equine Vet Sci 104:103688. 10.1016/j.jevs.2021.103688 10.1016/j.jevs.2021.103688 34416995

[CR61] Galiazzo G, De Silva M, Giancola F, Rinnovati R, Peli A, Chiocchetti R (2022) Cellular distribution of cannabinoid-related receptors TRPV1, PPAR-gamma, GPR55 and GPR3 in the equine cervical dorsal root ganglia. Equine Vet J 54:788–798. 10.1111/evj.13499 10.1111/evj.13499 PMC9293124 34418142

[CR62] Gamble L-J, Boesch JM, Frye CW, Schwark WS, Mann S, Wolfe L, Brown H, Berthelsen ES, Wakshlag JJ (2018) Pharmacokinetics, safety, and clinical efficacy of cannabidiol treatment in osteoarthritic dogs. Front Vet Sci 5:165. 10.3389/fvets.2018.00165 30083539 10.3389/fvets.2018.00165 PMC6065210

[CR63] Garcia GA, Kube S, Carrera-Justiz S, Tittle D, Wakshlag JJ (2022) Safety and efficacy of cannabidiol-cannabidiolic acid rich hemp extract in the treatment of refractory epileptic seizures in dogs. Front Vet Sci 9:939966. 10.3389/fvets.2022.939966 35967998 10.3389/fvets.2022.939966 PMC9372618

[CR64] Gebremedhin D, Lange AR, Campbell WB, Hillard CJ, Harder DR (1999) Cannabinoid CB1 receptor of cat cerebral arterial muscle functions to inhibit L-type ca ^2 + channel current. Am J Physiol Heart Circ Physiol 276:H2085–H2093. 10.1152/ajpheart.1999.276.6.H2085 10.1152/ajpheart.1999.276.6.H2085 10362691

[CR65] Gomez-Canas M, Rodrıguez-Cueto C, Satta V, Hernandez-Fisac I, Navarro E, Fernandez-Ruiz J (2023) Endocannabinoid-binding receptors as drug targets. Methods Mol Biol 2576:67–94. 10.1007/978-1-0716-2728-0_6 10.1007/978-1-0716-2728-0_6 36152178

[CR66] Gutierre E, Crosignani N, García-Carnelli C, Di Mateo A, Recchi L (2023) A case report of CBD and THC as analgesic therapy in a cat with chronic osteoarthritic pain. Vet Med Sci 9:1021–1025. 10.1002/vms3.1057 10.1002/vms3.1057 PMC10188064 37002652

[CR67] Hannon MB, Deabold KA, Talsma BN, Lyubimov A, Iqbal A, Zakharov A, Gamble LJ, Wakshlag JJ (2020) Serum cannabidiol, tetrahydrocannabinol (THC), and their native acid derivatives after transdermal application of a low-THC Cannabis sativa extract in beagles. J Vet Pharmacol Ther 43:508–511. 10.1111/jvp.12896 32735381 10.1111/jvp.12896

[CR68] Hasckel Gewehr JL, Enzele ML, Freiria LM, Nunes MM, Spengler J, Dondoerfer Teixeira AP, Amazonas E, Padilha S, V (2024) Full spectrum cannabidiol-rich extract reduced propofol dosage required for anesthetic induction in dogsa pilot study. Front Vet Sci 11:1352314. 10.3389/fvets.2024.1352314 38645644 10.3389/fvets.2024.1352314 PMC11026717

[CR69] Hegde VL, Singh UP, Nagarkatti PS, Nagarkatti M (2015) Critical role of mast cells and peroxisome proliferator-activated receptor γ in the induction of myeloid-derived suppressor cells by marijuana cannabidiol in vivo. J Immunol 194:5211–5222. 10.4049/JIMMUNOL.1401844 25917103 10.4049/jimmunol.1401844 PMC4433789

[CR70] Huang J, Korsunsky A, Yazdani M, Chen J (2024) Targeting TRP channels: recent advances in structure, ligand binding, and molecular mechanisms. Front Mol Neurosci 16. 10.3389/FNMOL.2023.1334370 10.3389/fnmol.2023.1334370 PMC10808746 38273937

[CR71] Hunt ABG, Flint HE, Logan DW, King T (2023) A single dose of cannabidiol (CBD) positively influences measures of stress in dogs during separation and car travel. Front Vet Sci 10:1112604. 10.3389/fvets.2023.1112604 10.3389/fvets.2023.1112604 PMC9992179 36908527

[CR72] Interlandi C, Tabbì M, Di Pietro S, DAngelo F, Costa GL, Arfuso F, Giudice E, Licata P, Macrì D, Crupi R, Gugliandolo E (2024) Improved quality of life and pain relief in mature horses with osteoarthritis after oral transmucosal cannabidiol oil administration as part of an analgesic regimen. Front Vet Sci 11:1341396. 10.3389/fvets.2024.1341396 10.3389/fvets.2024.1341396 PMC10876772 38379920

[CR73] Irving A, Abdulrazzaq G, Chan SLF, Penman J, Harvey J, Alexander SPH (2017) Cannabinoid receptor-related orphan G protein-coupled receptors. Adv Pharmacol 80:223–247. 10.1016/BS.APHA.2017.04.004 10.1016/bs.apha.2017.04.004 28826536

[CR74] Jukier T, Cruz-Espindola C, Martin D, Boothe DM (2023) Disposition of a single oral dose of a cannabidiol medication in healthy cats. Front Vet Sci 10:1181517. 10.3389/fvets.2023.1181517 10.3389/fvets.2023.1181517 PMC10251743 37303724

[CR75] Kathmann M, Flau K, Redmer A, Tränkle C, Schlicker E (2006) Cannabidiol is an allosteric modulator at mu- and delta-opioid receptors. Naunyn Schmiedebergs Arch Pharmacol 372:354–361. 10.1007/s00210-006-0033-x 16489449 10.1007/s00210-006-0033-x

[CR76] Khosropoor S, Alavi MS, Etemad L, Roohbakhsh A (2023) Cannabidiol goes nuclear: the role of PPARγ. Phytomedicine 114:154771. 10.1016/j.phymed.2023.154771 36965374 10.1016/j.phymed.2023.154771

[CR77] Kibret BG, Canseco-Alba A, Onaivi ES, Engidawork E (2023) Crosstalk between the endocannabinoid and mid-brain dopaminergic systems: implication in dopamine dysregulation. Front Behav Neurosci 17. 10.3389/fnbeh.2023.1137957 10.3389/fnbeh.2023.1137957 PMC10061032 37009000

[CR78] Klatzkow S, Davis G, Shmalberg J, Gallastegui A, Miscioscia E, Tarricone J, Elam L, Johnson MD, Leonard KM, Wakshlag JJ (2023) Evaluation of the efficacy of a cannabidiol and cannabidiolic acid rich hemp extract for pain in dogs following a tibial plateau leveling osteotomy. Front Vet Sci 9:1036056. 10.3389/fvets.2022.1036056 10.3389/fvets.2022.1036056 PMC9846637 36686184

[CR79] Kleinhenz MD, Magnin G, Lin Z, Griffin J, Kleinhenz KE, Montgomery S, Curtis A, Martin M, Coetzee JF (2020) Plasma concentrations of eleven cannabinoids in cattle following oral administration of industrial hemp (Cannabis sativa). Sci Rep 10:12753. 10.1038/s41598-020-69768-4 10.1038/s41598-020-69768-4 PMC7391639 32728233

[CR80] Kleinhenz MD, Weeder M, Montgomery S, Martin M, Curtis A, Magnin G, Lin Z, Griffin J, Coetzee JF (2022) Short term feeding of industrial hemp with a high cannabidiolic acid (CBDA) content increases lying behavior and reduces biomarkers of stress and inflammation in Holstein steers. Sci Rep 12:3683. 10.1038/s41598-022-07795-z 10.1038/s41598-022-07795-z PMC8901777 35256692

[CR81] Kogan LR, Hellyer PW, Silcox S-, Schoenfeld-Tacher R (2019a) Canadian dog owners’ use and perceptions of cannabis products. Can Vet J 60:749–755 PMC6563876 31281193

[CR82] Kogan L, Schoenfeld-Tacher R, Hellyer P, Rishniw M (2019b) US veterinarians’ knowledge, experience, and perception regarding the use of cannabidiol for canine medical conditions. Front Vet Sci 5:338. 10.3389/fvets.2018.00338 10.3389/fvets.2018.00338 PMC6338022 30687726

[CR83] Kogan L, Hellyer P, Downing R (2020) The use of cannabidiol-rich hemp oil extract to treat canine osteoarthritis-related pain: a pilot study. AHVMA J 58:35–42

[CR84] Kostic D, Nowakowska M, Freundt Revilla J, Attig F, Rohn K, Gualtieri F, Baumgärtner W, Potschka H, Tipold A (2023) Hippocampal expression of the cannabinoid receptor type 1 in canine epilepsy. Sci Rep 13:3138. 10.1038/s41598-023-29868-3 10.1038/s41598-023-29868-3 PMC9950490 36823232

[CR85] Krebs GL, De Rosa DW, White DM, Blake BL, Dods KC, May CD, Tai ZX, Clayton EH, Lynch EE (2021) Intake, nutrient digestibility, rumen parameters, growth rate, carcase characteristics and cannabinoid residues of sheep fed pelleted rations containing hemp (Cannabis sativa L.) Stubble. Transl Anim Sci 5:txab213. 10.1093/tas/txab213 10.1093/tas/txab213 PMC8714185 34988375

[CR86] Kulpa JE, Paulionis LJ, Eglit GM, Vaughn DM (2021) Safety and tolerability of escalating cannabinoid doses in healthy cats. J Feline Med Surg 23:1162–1175. 10.1177/1098612X211004215 10.1177/1098612X211004215 PMC8637357 33769105

[CR87] Kumar V, Kumaresan A, Nag P, Kumar P, Datta TK, Baithalu RK, Mohanty TK (2018) Transcriptional abundance of type-1 endocannabinoid receptor (CB1) and fatty acid amide hydrolase (FAAH) in bull spermatozoa: relationship with field fertility. Theriogenology 114:252–257. 10.1016/j.theriogenology.2018.04.001 10.1016/j.theriogenology.2018.04.001 29660628

[CR88] Kupczyk P, Rykala M, Serek P, Pawlak A, Slowikowski B, Holysz M, Chodaczek G, Madej JP, Ziolkowski P, Niedzwiedz A (2022) The cannabinoid receptors system in horses: tissue distribution and cellular identification in skin. J Vet Intern Med 36:1508–1524. 10.1111/jvim.16467 10.1111/jvim.16467 PMC9308437 35801813

[CR89] Lachowicz J, Szopa A, Ignatiuk K, Świ ader K, Serefko A (2023) Zebrafish as an animal model in cannabinoid research. Int J Mol Sci 24:10455. 10.3390/ijms241310455 10.3390/ijms241310455 PMC10341922 37445631

[CR90] Łebkowska-Wieruszewska B, Stefanelli F, Chericoni S, Owen H, Poapolathep A, Lisowski A, Giorgi M (2019) Pharmacokinetics of Bedrocan^®^, a cannabis oil extract, in fasting and fed dogs: an explorative study. Res Vet Sci 123:26–28. 10.1016/j.rvsc.2018.12.003 10.1016/j.rvsc.2018.12.003 30580232

[CR91] Leise JM, Leatherwood JL, Paris BL, Walter KW, George JM, Martinez RE, Glass KP, Lo C-P, Mays TP, Wickersham TA (2023) Evaluation of an oral supplemental cannabidiol product for acceptability and performance in mature horses. Animals 13:245. 10.3390/ani13020245 10.3390/ani13020245 PMC9854761 36670785

[CR92] Lile JA, Kelly TH, Hays LR (2014) Separate and combined effects of the GABAA positive allosteric modulator diazepam and ∆9-THC in humans discriminating ∆9-THC. Drug Alcohol Depend 143:141–148. 10.1016/j.drugalcdep.2014.07.016 25124305 10.1016/j.drugalcdep.2014.07.016 PMC4167716

[CR93] Lim J, Squire E, Jung K-M (2023) Phytocannabinoids, the endocannabinoid system and male reproduction. World J Mens Health 41:1. 10.5534/wjmh.220132 36578200 10.5534/wjmh.220132 PMC9826913

[CR94] Lima TdeM, Santiago NR, Alves ECR, Chaves DS, de Visacri A, M.B (2022) Use of cannabis in the treatment of animals: a systematic review of randomized clinical trials. Anim Health Res Rev 23:25–38. 10.1017/S1466252321000189 35703023 10.1017/S1466252321000189

[CR95] Limsuwan S, Phonsatta N, Panya A, Asasutjarit R, Tansakul N (2024) Pharmacokinetics behavior of four cannabidiol preparations following single oral administration in dogs. Front Vet Sci 11:1389810. 10.3389/fvets.2024.1389810 38725584 10.3389/fvets.2024.1389810 PMC11080651

[CR96] Loewinger M, Wakshlag JJ, Bowden D, Peters-Kennedy J, Rosenberg A (2022) The effect of a mixed cannabidiol and cannabidiolic acid based oil on client‐owned dogs with atopic dermatitis. Vet Dermatol 33:329. 10.1111/vde.13077 35644533 10.1111/vde.13077 PMC9543248

[CR97] Lucas CJ, Galettis P, Schneider J (2018) The pharmacokinetics and the pharmacodynamics of cannabinoids. Br J Clin Pharmacol 84:2477–2482. 10.1111/bcp.13710 30001569 10.1111/bcp.13710 PMC6177698

[CR98] Lutz B (2020) Neurobiology of cannabinoid receptor signaling. Dialogues Clin Neurosci 22:207–222. 10.31887/DCNS.2020.22.3/blutz 33162764 10.31887/DCNS.2020.22.3/blutz PMC7605026

[CR99] Lyons C, McEwan K, Munn-Patterson M, Vuong S, Alcorn J, Chicoine A (2024) Pharmacokinetic of two oral doses of a 1:20 THC:CBD cannabis herbal extract in cats. Front Vet Sci 11:1352495. 10.3389/fvets.2024.1352495 38585296 10.3389/fvets.2024.1352495 PMC10996858

[CR100] Maccarrone M, Marzo V, Di, Gertsch J, Grether U, Howlett AC, Hua T, Makriyannis A, Piomelli D, Ueda N, van der Stelt M (2023) Goods and bads of the endocannabinoid system as a therapeutic target: lessons learned after 30 years. Pharmacol Rev 75:885–958. 10.1124/PHARMREV.122.000600 37164640 10.1124/pharmrev.122.000600 PMC10441647

[CR101] Mariga C, Souza Silva Mateus AL, dos Santos Dullius ÂI, da Silva AP, Martins Flores M, Soares V, Amazonas A, Filho ETLP, S (2023) Dermatological evaluation in dogs with atopic dermatitis treated with full-spectrum high cannabidiol oil: a pre study part 1. Front Vet Sci 10. 10.3389/fvets.2023.1285384 10.3389/fvets.2023.1285384 PMC10644039 38026679

[CR102] Martello E, Bigliati M, Bisanzio D, Biasibetti E, Dosio F, Pastorino D, Nardi M, De, Bruni N (2019) Effects on pain and mobility of a new diet supplement in dogs with osteoarthritis: a pilot study. Ann Clin Lab Res 7:304

[CR103] Martinez Naya N, Kelly J, Corna G, Golino M, Abbate A, Toldo S (2023) Molecular and cellular mechanisms of action of cannabidiol. Molecules 28:5980. 10.3390/molecules28165980 37630232 10.3390/molecules28165980 PMC10458707

[CR104] Martins AM, Gomes AL, Vilas Boas I, Marto J, Ribeiro HM (2022) Cannabis-based products for the treatment of skin inflammatory diseases: a timely review. Pharmaceuticals (Basel) 15:210. 10.3390/ph15020210. Erratum in: Pharmaceuticals (Basel). Jul 11;15(7):849. 10.3390/ph15070849 10.3390/ph15020210 PMC8878527 35215320

[CR105] Masataka N (2024) Possible effects of cannabidiol (CBD) administration on the vocal activity of healthy domestic dogs upon their temporary separation from caregivers. Heliyon 10:e25548. 10.1016/j.heliyon.2024.e25548 10.1016/j.heliyon.2024.e25548 PMC10845910 38322918

[CR106] McGrath S, Bartner LR, Rao S, Kogan LR, Hellyer PW (2018) A report of adverse effects associated with the administration of cannabidiol in healthy dogs. AHVMA J 52:34–38

[CR107] McGrath S, Bartner LR, Rao S, Packer RA, Gustafson DL (2019) Randomized blinded controlled clinical trial to assess the effect of oral cannabidiol administration in addition to conventional antiepileptic treatment on seizure frequency in dogs with intractable idiopathic epilepsy. J Am Vet Med Assoc 254:1301–1308. 10.2460/javma.254.11.1301 10.2460/javma.254.11.1301 31067185

[CR108] McIver V, Tsang A, Symonds N, Perkins N, Uquillas E, Dart C, Jeffcott L, Dart A (2020) Effects of topical treatment of cannabidiol extract in a unique manuka factor 5 manuka honey carrier on second intention wound healing on equine distal limb wounds: a preliminary study. Aust Vet J 98:250–255. 10.1111/avj.12932 10.1111/avj.12932 32096215

[CR109] McPartland JM, Duncan M, Di Marzo V, Pertwee RG (2015) Are cannabidiol and ∆(9) -tetrahydrocannabivarin negative modulators of the endocannabinoid system? A systematic review. Br J Pharmacol 172:737–753. 10.1111/bph.12944 25257544 10.1111/bph.12944 PMC4301686

[CR110] Mechoulam R (2023) A delightful trip along the pathway of cannabinoid and endocannabinoid chemistry and pharmacology. Annu Rev Pharmacol Toxicol 63:1–13. 10.1146/annurev-pharmtox-051921-083709 10.1146/annurev-pharmtox-051921-083709 35850522

[CR111] Mechoulam R, Hanuš L (2002) Cannabidiol: an overview of some chemical and pharmacological aspects. Part I: chemical aspects. Chem Phys Lipids 121:35–43. 10.1016/S0009-3084(02)00144-5 12505688 10.1016/s0009-3084(02)00144-5

[CR112] Mejia S, Duerr FM, Griffenhagen G, McGrath S (2021) Evaluation of the effect of cannabidiol on naturally occurring osteoarthritis-associated pain: a pilot study in dogs. J Am Anim Hosp Assoc 57:81–90. 10.5326/JAAHA-MS-7119 10.5326/JAAHA-MS-7119 33450016

[CR113] Mercati F, DallAglio C, Pascucci L, Boiti C, Ceccarelli P (2012) Identification of cannabinoid type 1 receptor in dog hair follicles. Acta Histochem 114:68–71. 10.1016/j.acthis.2011.01.003 21414652 10.1016/j.acthis.2011.01.003

[CR114] Meyer K, Hayman K, Baumgartner J, Gorden PJ (2022) Plasma pharmacokinetics of cannabidiol following oral administration of cannabidiol oil to dairy calves. Front Vet Sci 9:789495. 10.3389/fvets.2022.789495 35141311 10.3389/fvets.2022.789495 PMC8818876

[CR115] Miagkoff L, Girard CA, St-Jean G, Richard H, Beauchamp G, Laverty S (2023) Cannabinoid receptors are expressed in equine synovium and upregulated with synovitis. Equine Vet J 55:681–695. 10.1111/evj.13860 35836386 10.1111/evj.13860

[CR116] Migliaro M, Ruiz-Contreras AE, Herrera-Solís A, Méndez-Díaz M, Prospéro-García OE (2023) Endocannabinoid system and aggression across animal species. Neurosci Biobehav Rev 153:105375. 10.1016/j.neubiorev.2023.105375 37643683 10.1016/j.neubiorev.2023.105375

[CR117] Millar SA, Maguire RF, Yates AS, OSullivan SE (2020) Towards better delivery of cannabidiol (CBD). Pharmaceuticals 13:219. 10.3390/ph13090219 32872355 10.3390/ph13090219 PMC7558665

[CR118] Miragliotta V, Ricci PL, Albanese F, Pirone A, Tognotti D, Abramo F (2018) Cannabinoid receptor types 1 and 2 and peroxisome proliferator-activated receptor‐α: distribution in the skin of clinically healthy cats and cats with hypersensitivity dermatitis. Vet Dermatol 29:316. 10.1111/vde.12658 10.1111/vde.12658 29920828

[CR119] Miranda-Cortés A, Mota-Rojas D, Crosignani-Outeda N, Casas-Alvarado A, Martínez-Burnes J, Olmos-Hernández A, Mora-Medina P, Verduzco-Mendoza A, Hernández-Ávalos I (2023) The role of cannabinoids in pain modulation in companion animals. Front Vet Sci 9:1050884. 10.3389/fvets.2022.1050884 10.3389/fvets.2022.1050884 PMC9848446 36686189

[CR120] Mogi C, Fukuyama T (2019) Cannabidiol as a potential anti-epileptic dietary supplement in dogs with suspected epilepsy: three case reports. Pet Behav Sci 11–16. 10.21071/pbs.v0i7.11800

[CR121] Mogi C, Yoshida M, Kawano K, Fukuyama T, Arai T (2022) Effects of cannabidiol without delta-9-tetrahydrocannabinol on canine atopic dermatitis: a retrospective assessment of 8 cases. Can Veterinary J 63:423 PMC8922375 35368394

[CR122] Morales P, Reggio PH (2017) An update on non-CB1, Non-CB2 cannabinoid related g-protein-coupled receptors. Cannabis Cannabinoid Res 2:265–273. 10.1089/can.2017.0036 29098189 10.1089/can.2017.0036 PMC5665501

[CR123] Morales P, Lago-Fernandez A, Hurst DP, Sotudeh N, Brailoiu E, Reggio PH, Abood ME, Jagerovic N (2020) Therapeutic exploitation of GPR18: beyond the cannabinoids? J Med Chem 63:14216–14227. 10.1021/acs.jmedchem.0c00926 10.1021/acs.jmedchem.0c00926 PMC7949482 32914978

[CR124] Morris EM, Kitts-Morgan SE, Spangler DM, McLeod KR, Costa JHC, Harmon DL (2020) The impact of feeding Cannabidiol (CBD) containing treats on canine response to a noise-induced fear response test. Front Vet Sci 7:569565. 10.3389/fvets.2020.569565 10.3389/fvets.2020.569565 PMC7537661 33195551

[CR125] Morris EM, Kitts-Morgan SE, Spangler DM, Gebert J, Vanzant ES, McLeod KR, Harmon DL (2021) Feeding cannabidiol (CBD)-containing treats did not affect canine daily voluntary activity. Front Vet Sci 8:645667. 10.3389/fvets.2021.645667 10.3389/fvets.2021.645667 PMC8118201 33996972

[CR126] Nabissi M, Morelli MB, Amantini C, Liberati S, Santoni M, Ricci-Vitiani L, Pallini R, Santoni G (2015) Cannabidiol stimulates Aml-1a-dependent glial differentiation and inhibits glioma stem-like cells proliferation by inducing autophagy in a TRPV2-dependent manner. Int J Cancer 137:1855–1869. 10.1002/IJC.29573 10.1002/ijc.29573 25903924

[CR127] O’Sullivan SE (2016) An update on PPAR activation by cannabinoids. Br J Pharmacol 173:1899–1910. 10.1111/bph.13497 27077495 10.1111/bph.13497 PMC4882496

[CR128] Panda C, Rathinasabapathy T, Metzger B, Dodson S, Hanson D, Griffiths J, Komarnytsky S (2024) Efficacy and tolerability of full spectrum hemp oil in dogs living with pain in common household settings. Front Vet Sci 11:1384168. 10.3389/fvets.2024.1384168 10.3389/fvets.2024.1384168 PMC11272626 39071787

[CR129] Patikorn C, Nerapusee O, Soontornvipart K, Lawonyawut K, Musikpodok K, Waleethanaphan K, Anantachoti P (2023) Efficacy and safety of cannabidiol for the treatment of canine osteoarthritis: a systematic review and meta-analysis of animal intervention studies. Front Vet Sci 10. 10.3389/fvets.2023.1248417 10.3389/fvets.2023.1248417 PMC10540436 37781283

[CR130] Pérez-Acevedo A, Busardò F, Pacifici R, Mannocchi G, Gottardi M, Poyatos L, Papaseit E, Pérez-Mañá C, Martin S, Di Trana A, Pichini S, Farré M (2020) Disposition of cannabidiol metabolites in serum and urine from healthy individuals treated with pharmaceutical preparations of medical cannabis. Pharmaceuticals 13:459. 10.3390/ph13120459 10.3390/ph13120459 PMC7763054 33322849

[CR131] Perucca E, Bialer M (2020) Critical aspects affecting cannabidiol oral bioavailability and metabolic elimination, and related clinical implications. CNS Drugs 34:795–800. 10.1007/s40263-020-00741-5 32504461 10.1007/s40263-020-00741-5

[CR132] Peyravian N, Deo S, Daunert S, Jimenez JJ (2020) Cannabidiol as a novel therapeutic for immune modulation. Immunotargets Ther 9:131–140. 10.2147/ITT.S263690 10.2147/ITT.S263690 PMC7445536 32903924

[CR133] Pirone A, Lenzi C, Coli A, Giannessi E, Stornelli MR, Miragliotta V (2015) Preferential epithelial expression of type-1 cannabinoid receptor (CB1R) in the developing canine embryo. Springerplus 4:804. 10.1186/s40064-015-1616-0 10.1186/s40064-015-1616-0 PMC4688286 26702393

[CR134] Pirone A, Cantile C, Miragliotta V, Lenzi C, Giannessi E, Cozzi B (2016) Immunohistochemical distribution of the cannabinoid receptor 1 and fatty acid amide hydrolase in the dog claustrum. J Chem Neuroanat 74:21–27. 10.1016/j.jchemneu.2016.02.002 10.1016/j.jchemneu.2016.02.002 26907575

[CR135] Pirone A, Lenzi C, Briganti A, Abbate F, Levanti M, Abramo F, Miragliotta V (2017) Spatial distribution of cannabinoid receptor 1 and fatty acid amide hydrolase in the cat ovary and oviduct. Acta Histochem 119:417–422. 10.1016/j.acthis.2017.04.007 10.1016/j.acthis.2017.04.007 28478955

[CR136] Pirone A, Lazzarini G, Lenzi C, Giannessi E, Miragliotta V (2020) Immunolocalization of cannabinoid receptor 1 (CB1), monoglyceride lipase (MGL) and fatty-acid amide hydrolase 1 (FAAH) in the pig claustrum. J Chem Neuroanat 109:101843. 10.1016/j.jchemneu.2020.101843 10.1016/j.jchemneu.2020.101843 32599254

[CR137] Polidoro G, Galiazzo G, Giancola F, Papadimitriou S, Kouki M, Sabattini S, Rigillo A, Chiocchetti R (2021) Expression of cannabinoid and cannabinoid-related receptors in the oral mucosa of healthy cats and cats with chronic gingivostomatitis. J Feline Med Surg 23:679–691. 10.1177/1098612X20970510 10.1177/1098612X20970510 PMC10812186 33174485

[CR138] Polidoro D, Temmerman R, Devreese M, Charalambous M, Ham L, Van, Cornelis I, Broeckx BJG, Mandigers PJJ, Fischer A, Storch J, Bhatti SFM (2022) Pharmacokinetics of cannabidiol following intranasal, intrarectal, and oral administration in healthy dogs. Front Vet Sci 9:899940. 10.3389/fvets.2022.899940 10.3389/fvets.2022.899940 PMC9215213 35754531

[CR139] Rajan TS, Giacoppo S, Iori R, De Nicola GR, Grassi G, Pollastro F, Bramanti P, Mazzon E (2016) Anti-inflammatory and antioxidant effects of a combination of cannabidiol and moringin in LPS-stimulated macrophages. Fitoterapia 112:104–115. 10.1016/J.FITOTE.2016.05.008 10.1016/j.fitote.2016.05.008 27215129

[CR140] Rinaldi V, Boari A, Ressel L, Bongiovanni L, Crisi PE, Cabibbo E, Finotello R (2022) Expression of cannabinoid receptors CB1 and CB2 in canine cutaneous mast cell tumours. Res Vet Sci 152:530–536. 10.1016/j.rvsc.2022.09.013 10.1016/j.rvsc.2022.09.013 36179546

[CR141] Rodríguez-Muñoz M, Onetti Y, Cortés-Montero E, Garzón J, Sánchez-Blázquez P (2018) Cannabidiol enhances morphine antinociception, diminishes NMDA-mediated seizures and reduces stroke damage via the sigma 1 receptor. Mol Brain 11:51. 10.1186/s13041-018-0395-2 10.1186/s13041-018-0395-2 PMC6142691 30223868

[CR142] Rooney TA, Carpenter JW, KuKanich B, Gardhouse SM, Magnin GC, Tully TN (2022) Feeding decreases the oral bioavailability of cannabidiol and cannabidiolic acid in hemp oil in New Zealand White rabbits (Oryctolagus cuniculus). Am J Vet Res 83. ajvr.22.01.0006 10.2460/ajvr.22.01.0006 35947680

[CR143] Rozental AJ, Gustafson DL, Kusick BR, Bartner LR, Castro SC, McGrath S (2023a) Pharmacokinetics of escalating single-dose administration of cannabidiol to cats. J Vet Pharmacol Ther 46:25–33. 10.1111/jvp.13100 10.1111/jvp.13100 PMC10092881 36300854

[CR144] Rozental AJ, Weisbeck BG, Corsato Alvarenga I, Gustafson DL, Kusick BR, Rao S, Bartner LR, McGrath S (2023b) The efficacy and safety of cannabidiol as adjunct treatment for drug-resistant idiopathic epilepsy in 51 dogs: a double‐blinded crossover study. J Vet Intern Med 37:2291–2300. 10.1111/jvim.16912 10.1111/jvim.16912 PMC10658598 37889215

[CR145] Ruel HLM, Monteiro BP, Blais E, Richard H, St-Jean G, Laverty S, Steagall PV (2022) Immunohistochemical localization of cannabinoid receptor type I in the feline synovial membrane with and without degenerative lesions. in Proceedings of the AVA Spring Meeting 2022. pp. 116, Association of Veterinary Anaesthetists (AVA) Spring Meeting (2022), Nafplio, Greece, 18/05/22

[CR146] Ruffolo G, Gaeta A, Cannata B, Pinzaglia C, Aronica E, Morano A, Cifelli P, Palma E (2022) GABAergic neurotransmission in human tissues is modulated by cannabidiol. Life 12:2042. 10.3390/life12122042 10.3390/life12122042 PMC9786817 36556407

[CR147] Ryan D, McKemie DS, Kass PH, Puschner B, Knych HK (2021) Pharmacokinetics and effects on arachidonic acid metabolism of low doses of cannabidiol following oral administration to horses. Drug Test Anal 13:1305–1317. 10.1002/dta.3028 10.1002/dta.3028 33723919

[CR148] Rybarczyk A, Majchrzak-Celińska A, Krajka-Kuźniak V (2023) Targeting Nrf2 signaling pathway in cancer prevention and treatment: the role of cannabis compounds. Antioxid (Basel) 12. 10.3390/ANTIOX12122052 10.3390/antiox12122052 PMC10740807 38136172

[CR149] Ryberg E, Larsson N, Sjögren S, Hjorth S, Hermansson N-O, Leonova J, Elebring T, Nilsson K, Drmota T, Greasley PJ (2007) The orphan receptor GPR55 is a novel cannabinoid receptor. Br J Pharmacol 152:1092–1101. 10.1038/sj.bjp.0707460 10.1038/sj.bjp.0707460 PMC2095107 17876302

[CR150] Samara E, Bialer M, Mechoulam R (1988) Pharmacokinetics of cannabidiol in dogs. Drug Metab Dispos 16:469–472 2900742

[CR151] Samara E, Bialer M, Harvey DJ (1990) Pharmacokinetics of urinary metabolites of cannabidiol in the dog. Biopharm Drug Dispos 11:785–795. 10.1002/bdd.2510110906 10.1002/bdd.2510110906 2271754

[CR152] Sánchez De Medina, A., Serrano-Rodríguez, J.M., Díez De Castro, E., García-Valverde, M.T., Saitua, A., Becero, M., Muñoz, A., Ferreiro-Vera, C., Sánchez De Medina, V., 2023. Pharmacokinetics and oral bioavailability of cannabidiol in horses after intravenous and oral administration with oil and micellar formulations. Equine Vet J 55, 1094–1103. Equine Vet J 55:1094–1103. 10.1111/evj.13923 10.1111/evj.13923 36624043

[CR153] Schaefer N, Wojtyniak J-G, Kettner M, Schlote J, Laschke MW, Ewald AH, Lehr T, Menger MD, Maurer HH, Schmidt PH (2016) Pharmacokinetics of (synthetic) cannabinoids in pigs and their relevance for clinical and forensic toxicology. Toxicol Lett 253:7–16. 10.1016/j.toxlet.2016.04.021 10.1016/j.toxlet.2016.04.021 27113702

[CR154] Schaefer N, Kettner M, Laschke MW, Schlote J, Ewald AH, Menger MD, Maurer HH, Schmidt PH (2017) Distribution of synthetic cannabinoids JWH-210, RCS-4 and ∆ 9-Tetrahydrocannabinol after intravenous administration to pigs. Curr Neuropharmacol 15. 10.2174/1570159X15666161111114214 10.2174/1570159X15666161111114214 PMC5771047 27834143

[CR155] Senn L, Cannazza G, Biagini G (2020) Receptors and channels possibly mediating the effects of phytocannabinoids on seizures and epilepsy. Pharmaceuticals (Basel Switzerland) 13:174. 10.3390/ph13080174 10.3390/ph13080174 PMC7463541 32751761

[CR156] Sermet S, Li J, Bach A, Crawford RB, Kaminski NE (2021) Cannabidiol selectively modulates interleukin (IL)-1β and IL-6 production in toll-like receptor activated human peripheral blood monocytes. Toxicology 464. 10.1016/J.TOX.2021.153016 10.1016/j.tox.2021.153016 PMC9196914 34740670

[CR157] Shilo-Benjamini Y, Cern A, Zilbersheid D, Hod A, Lavy E, Barasch D, Barenholz Y (2022) A case report of subcutaneously injected liposomal cannabidiol formulation used as a compassion therapy for pain management in a dog. Front Vet Sci 9:892306. 10.3389/fvets.2022.892306 10.3389/fvets.2022.892306 PMC9097221 35573415

[CR158] Shilo-Benjamini Y, Lavy E, Yair N, Milgram J, Zilbersheid D, Hod A, Barasch D, Abu Ahmad W, Cern A, Barenholz Y (2023) Therapeutic efficacy and pharmacokinetics of liposomal-cannabidiol injection: a pilot clinical study in dogs with naturally-occurring osteoarthritis. Front Vet Sci 10. 10.3389/fvets.2023.1224452 10.3389/fvets.2023.1224452 PMC10481162 37680386

[CR159] Silver RJ (2019) The endocannabinoid system of animals. Animals 9:686. 10.3390/ani9090686 10.3390/ani9090686 PMC6770351 31527410

[CR160] Simpson AC, Bradley CW, Schissler JR (2020) Probable cutaneous adverse drug reaction due to a cannabidiol-containing hemp oil product in a dog. Vet Dermatol 31:404. 10.1111/vde.12876 10.1111/vde.12876 32735064

[CR161] Sitovs A, Logviss K, Lauberte L, Mohylyuk V (2024) Oral delivery of cannabidiol: revealing the formulation and absorption challenges. J Drug Deliv Sci Technol 92:105316. 10.1016/j.jddst.2023.105316

[CR162] Soga T, Ohishi T, Matsui T, Saito T, Matsumoto M, Takasaki J, Matsumoto SI, Kamohara M, Hiyama H, Yoshida S, Momose K, Ueda Y, Matsushime H, Kobori M, Furuichi K (2005) Lysophosphatidylcholine enhances glucose-dependent insulin secretion via an orphan G-protein-coupled receptor. Biochem Biophys Res Commun 326:744–751. 10.1016/J.BBRC.2004.11.120 10.1016/j.bbrc.2004.11.120 15607732

[CR163] Sosa-Higareda M, Guzman DS-M, Knych H, Lyubimov A, Zakharov A, Gomez B, Beaufrère H (2023) Twice-daily oral administration of a cannabidiol and cannabidiolic acidrich hemp extract was well tolerated in orange-winged Amazon parrots (Amazona amazonica) and has a favorable pharmacokinetic profile. Am J Vet Res 1–11. 10.2460/ajvr.22.11.0197 10.2460/ajvr.22.11.0197 36795552

[CR164] St. Blanc MP, Chapman AM, Keowen ML, Garza F, Liu C-C, Gray L, Andrews FM (2022) Effects of a supplement containing cannabidiol (CBD) on sedation and ataxia scores and health. J Equine Vet Sci 117:104085. 10.1016/j.jevs.2022.104085 10.1016/j.jevs.2022.104085 35882292

[CR165] Stanzani A, Galiazzo G, Giancola F, Tagliavia C, De Silva M, Pietra M, Fracassi F, Chiocchetti R (2020) Localization of cannabinoid and cannabinoid related receptors in the cat gastrointestinal tract. Histochem Cell Biol 153:339–356. 10.1007/s00418-020-01854-0 10.1007/s00418-020-01854-0 32095931

[CR166] Starkus J, Jansen C, Shimoda LMN, Stokes AJ, Small-Howard AL, Turner H (2019) Diverse TRPV1 responses to cannabinoids. Channels (Austin) 13:172–191. 10.1080/19336950.2019.1619436 31096838 10.1080/19336950.2019.1619436 PMC6557596

[CR167] Stevens SA, Krebs GL, Scrivener CJ, Noble GK, Blake BL, Dods KC, May CD, Tai ZX, Clayton EH, Lynch EE, Johnson KN (2022) Nutrient digestibility, rumen parameters, and (cannabinoid) residues in sheep fed a pelleted diet containing green hemp (Cannabis sativa L.) biomass. Transl Anim Sci 6:txac141. 10.1093/tas/txac141 10.1093/tas/txac141 PMC9661295 36381952

[CR168] Stincic TL, Hyson RL (2008) Localization of CB1 cannabinoid receptor mRNA in the brain of the chick (Gallus Domesticus). Brain Res 1245:61–73. 10.1016/j.brainres.2008.09.037 10.1016/j.brainres.2008.09.037 PMC2636724 18835551

[CR169] Taylor L, Gidal B, Blakey G, Tayo B, Morrison G (2018) A phase I, randomized, double-blind, placebo-controlled, single ascending dose, multiple dose, and food effect trial of the safety, tolerability and pharmacokinetics of highly purified cannabidiol in healthy subjects. CNS Drugs 32:1053–1067. 10.1007/s40263-018-0578-5 10.1007/s40263-018-0578-5 PMC6223703 30374683

[CR170] Thomson ACS, McCarrel TM, Zakharov A, Gomez B, Lyubimov A, Schwark WS, Mallicote MF, Portela DA, Bisiau AL, Wakshlag JJ (2024) Pharmacokinetics and tolerability of single-dose enteral cannabidiol and cannabidiolic acid rich hemp in horses (Equus caballus). Front Vet Sci 11:1356463. 10.3389/fvets.2024.1356463 38681854 10.3389/fvets.2024.1356463 PMC11047043

[CR171] Tittle D, Wakshlag J, Schwark W, Lyubimov A, Zakharov A, Gomez B (2022) Twenty-four hour and one-week steady state pharmacokinetics of cannabinoids in two formulations of cannabidiol and cannabidiolic acid rich hemp in dogs. Med Res Arch 10. 10.18103/mra.v10i7.2907

[CR172] Toschi A, Galiazzo G, Piva A, Tagliavia C, Mazzuoli-Weber G, Chiocchetti R, Grilli E (2021) Cannabinoid and cannabinoid-related receptors in the myenteric plexus of the porcine ileum. Animals 11:263. 10.3390/ani11020263 10.3390/ani11020263 PMC7912003 33494452

[CR173] Turner SE, Knych HK, Adams AA (2022) Pharmacokinetics of cannabidiol in a randomized crossover trial in senior horses. Am J Vet Res 83. 10.2460/ajvr.22.02.0028. ajvr.22.02.0028 10.2460/ajvr.22.02.0028 35895770

[CR174] Turner S, Knych HK, Adams AA (2023) The effects of cannabidiol on immune function and health parameters in senior horses. Vet Immunol Immunopathol 257:110549. 10.1016/j.vetimm.2023.110549 10.1016/j.vetimm.2023.110549 36682327

[CR175] Vaughn D, Kulpa J, Paulionis L (2020) Preliminary investigation of the safety of escalating cannabinoid doses in healthy dogs. Front Vet Sci 7:51. 10.3389/fvets.2020.00051 32118071 10.3389/fvets.2020.00051 PMC7029731

[CR176] Vaughn DM, Paulionis LJ, Kulpa JE (2021) Randomized, placebo-controlled, 28-day safety and pharmacokinetics evaluation of repeated oral cannabidiol administration in healthy dogs. Am J Vet Res 82:405–416. 10.2460/ajvr.82.5.405 33904801 10.2460/ajvr.82.5.405

[CR177] Verrico CD, Wesson S, Konduri V, Hofferek CJ, Vazquez-Perez J, Blair E, Dunner K, Salimpour P, Decker WK, Halpert MM (2020) A randomized, double-blind, placebo-controlled study of daily cannabidiol for the treatment of canine osteoarthritis pain. Pain 161:2191–2202. 10.1097/j.pain.0000000000001896 32345916 10.1097/j.pain.0000000000001896 PMC7584779

[CR178] Vrechi TAM, Leão AHFF, Morais IBM, Abílio VC, Zuardi AW, Hallak JEC, Crippa JA, Bincoletto C, Ureshino RP, Smaili SS, Pereira GJS (2021) Cannabidiol induces autophagy via ERK1/2 activation in neural cells. Sci Rep 11:5434. 10.1038/S41598-021-84879-2 10.1038/s41598-021-84879-2 PMC7940388 33686185

[CR179] Wagner B, Gerletti P, Fürst P, Keuth O, Bernsmann T, Martin A, Schäfer B, Numata J, Lorenzen MC, Pieper R (2022) Transfer of cannabinoids into the milk of dairy cows fed with industrial hemp could lead to ∆9-THC exposure that exceeds acute reference dose. Nat Food 3:921–932. 10.1038/s43016-022-00623-7 10.1038/s43016-022-00623-7 37118216

[CR180] Wakshlag JJ, Schwark WS, Deabold KA, Talsma BN, Cital S, Lyubimov A, Iqbal A, Zakharov A (2020) Pharmacokinetics of cannabidiol, cannabidiolic acid, ∆9-Tetrahydrocannabinol, tetrahydrocannabinolic acid and related metabolites in canine serum after dosing with three oral forms of hemp extract. Front Vet Sci 7:505. 10.3389/fvets.2020.00505 10.3389/fvets.2020.00505 PMC7498943 33102539

[CR181] Wang T, Zakharov A, Gomez B, Lyubimov A, Trottier NL, Schwark WS, Wakshlag JJ (2022) Serum cannabinoid 24 h and 1 week steady state pharmacokinetic assessment in cats using a CBD/CBDA rich hemp paste. Front Vet Sci 9:895368. 10.3389/fvets.2022.895368 10.3389/fvets.2022.895368 PMC9355628 35937287

[CR182] Whyte LS, Ryberg E, Sims NA, Ridge SA, Mackie K, Greasley PJ, Ross RA, Rogers MJ (2009) The putative cannabinoid receptor GPR55 affects osteoclast function in vitro and bone mass in vivo. Proc Natl Acad Sci 106:16511–16516. 10.1073/pnas.0902743106 10.1073/pnas.0902743106 PMC2737440 19805329

[CR183] Williams MR, Holbrook TC, Maxwell L, Croft CH, Ientile MM, Cliburn K (2022) Pharmacokinetic evaluation of a cannabidiol supplement in horses. J Equine Vet Sci 110:103842. 10.1016/j.jevs.2021.103842 10.1016/j.jevs.2021.103842 34923070

[CR184] Wright J (2022) Evaluating the benefits of cannabidiol for analgesia following surgery for intervertebral disc herniation in dogs. Proceedings of the American College of Veterinary Internal Medicine Forum. American College of Veterinary Internal Medicine; 2022. In: American College of Veterinary Internal Medicine (ed) Proceedings of the American College of Veterinary Internal Medicine Forum

[CR185] Ye L, Cao Z, Wang W, Zhou N (2019) New insights in cannabinoid receptor structure and signaling. Curr Mol Pharmacol 12:239–248. 10.2174/1874467212666190215112036 10.2174/1874467212666190215112036 PMC6864585 30767756

[CR186] Yocom AF, O’Fallon ES, Gustafson DL, Contino EK (2022) Pharmacokinetics, safety, and synovial fluid concentrations of single- and multiple-dose oral administration of 1 and 3 mg/kg cannabidiol in horses. J Equine Vet Sci 113:103933. 10.1016/j.jevs.2022.103933 10.1016/j.jevs.2022.103933 35307550

[CR187] Zamith Cunha R, Salamanca G, Mille F, Delprete C, Franciosi C, Piva G, Gramenzi A, Chiocchetti R (2023a) Endocannabinoid system receptors at the hip and stifle joints of middle-aged dogs: a novel target for the therapeutic use of cannabis sativa extract in canine arthropathies. Animals 13:2833. 10.3390/ani13182833 10.3390/ani13182833 PMC10525782 37760233

[CR188] Zamith Cunha R, Semprini A, Salamanca G, Gobbo F, Morini M, Pickles KJ, Roberts V, Chiocchetti R (2023b) Expression of cannabinoid receptors in the trigeminal ganglion of the horse. Int J Mol Sci 24:15949. 10.3390/ijms242115949 10.3390/ijms242115949 PMC10648827 37958932

[CR189] Zamith Cunha R, Zannoni A, Salamanca G, De Silva M, Rinnovati R, Gramenzi A, Forni M, Chiocchetti R (2023c) Expression of cannabinoid (CB1 and CB2) and cannabinoid-related receptors (TRPV1, GPR55, and PPARα) in the synovial membrane of the horse metacarpophalangeal joint. Front Vet Sci 10:1045030. 10.3389/fvets.2023.1045030 10.3389/fvets.2023.1045030 PMC10020506 36937015

[CR190] Zou S, Kumar U (2018) Cannabinoid receptors and the endocannabinoid system: signaling and function in the central nervous system. Int J Mol Sci 19:833. 10.3390/ijms19030833 10.3390/ijms19030833 PMC5877694 29533978

